# Pre-analytical challenges from adsorptive losses associated with thiamine analysis

**DOI:** 10.1038/s41598-024-60910-0

**Published:** 2024-05-04

**Authors:** Katie A. Edwards, Eileen A. Randall, Patricia C. Wolfe, Clifford E. Kraft, Esther R. Angert

**Affiliations:** 1 Department of Pharmaceutical Sciences, Binghamton University, Binghamton, NY 13902 USA; 2 Department of Microbiology, Cornell University, Ithaca, NY 14853 USA; 3 Department of Natural Resources and the Environment, Cornell University, Ithaca, NY 14853 USA

**Keywords:** Analytical biochemistry, Isolation, separation and purification, Analytical chemistry, Medical and clinical diagnostics, Biochemistry

## Abstract

Thiamine (vitamin B1) is an essential vitamin serving in its diphosphate form as a cofactor for enzymes in the citric acid cycle and pentose-phosphate pathways. Its concentration reported in the pM and nM range in environmental and clinical analyses prompted our consideration of the components used in pre-analytical processing, including the selection of filters, filter apparatuses, and sample vials. The seemingly innocuous use of glass fiber filters, glass filter flasks, and glass vials, ubiquitous in laboratory analysis of clinical and environmental samples, led to marked thiamine losses. 19.3 nM thiamine was recovered from a 100 nM standard following storage in glass autosampler vials and only 1 nM of thiamine was obtained in the filtrate of a 100 nM thiamine stock passed through a borosilicate glass fiber filter. We further observed a significant shift towards phosphorylated derivatives of thiamine when an equimolar mixture of thiamine, thiamine monophosphate, and thiamine diphosphate was stored in glass (most notably non-silanized glass, where a reduction of 54% of the thiamine peak area was observed) versus polypropylene autosampler vials. The selective losses of thiamine could lead to errors in interpreting the distribution of phosphorylated species in samples. Further, some loss of phosphorylated thiamine derivatives selectively to amber glass vials was observed relative to other glass vials. Our results suggest the use of polymeric filters (including nylon and cellulose acetate) and storage container materials (including polycarbonate and polypropylene) for thiamine handling. Losses to cellulose nitrate and polyethersulfone filters were far less substantial than to glass fiber filters, but were still notable given the low concentrations expected in samples. Thiamine losses were negated when thiamine was stored diluted in trichloroacetic acid or as thiochrome formed in situ, both of which are common practices, but not ubiquitous, in thiamine sample preparation.

## Introduction

Thiamine (vitamin B1) serves in its diphosphate form as a cofactor for enzymes involved in carbohydrate metabolism and amino acid catabolism. As an essential cofactor, it is necessary for the health of all living organisms. Thiamine is found at low nM concentrations in human plasma while at low pM concentrations in environmental waters^1–5^. Competition for low levels of thiamine available in the environment is evident among microorganisms and is associated with mortality of commercially valuable fish, ruminant mammals and aquatic invertebrates^6,7^. Thiamine deficiency has been a long-standing concern in economically and ecologically important fisheries and is often associated with increased consumption of prey fish containing thiaminase, an enzyme that breaks down thiamine^8–10^. In humans, thiamine deficiency is commonly associated with dietary insufficiency or insufficient absorption in people with primarily rice-based diets or alcoholism^11,12^. While thiamine supplementation of foods is common, overt deficiency due to thiamine omission or breakdown in processed foods in consumers largely relying upon a singular dietary source (e.g., companion animals and infants) has been reported^13–15^. As thiamine and its breakdown products are increasingly subject to monitoring and study, consideration of pre-analytical characteristics is warranted. Within, we report substantial adsorptive losses of thiamine to commonly used storage vessels and filters, leading to concern that these losses may impact the interpretation of results in environmental and clinical samples.

**Figure 1:**
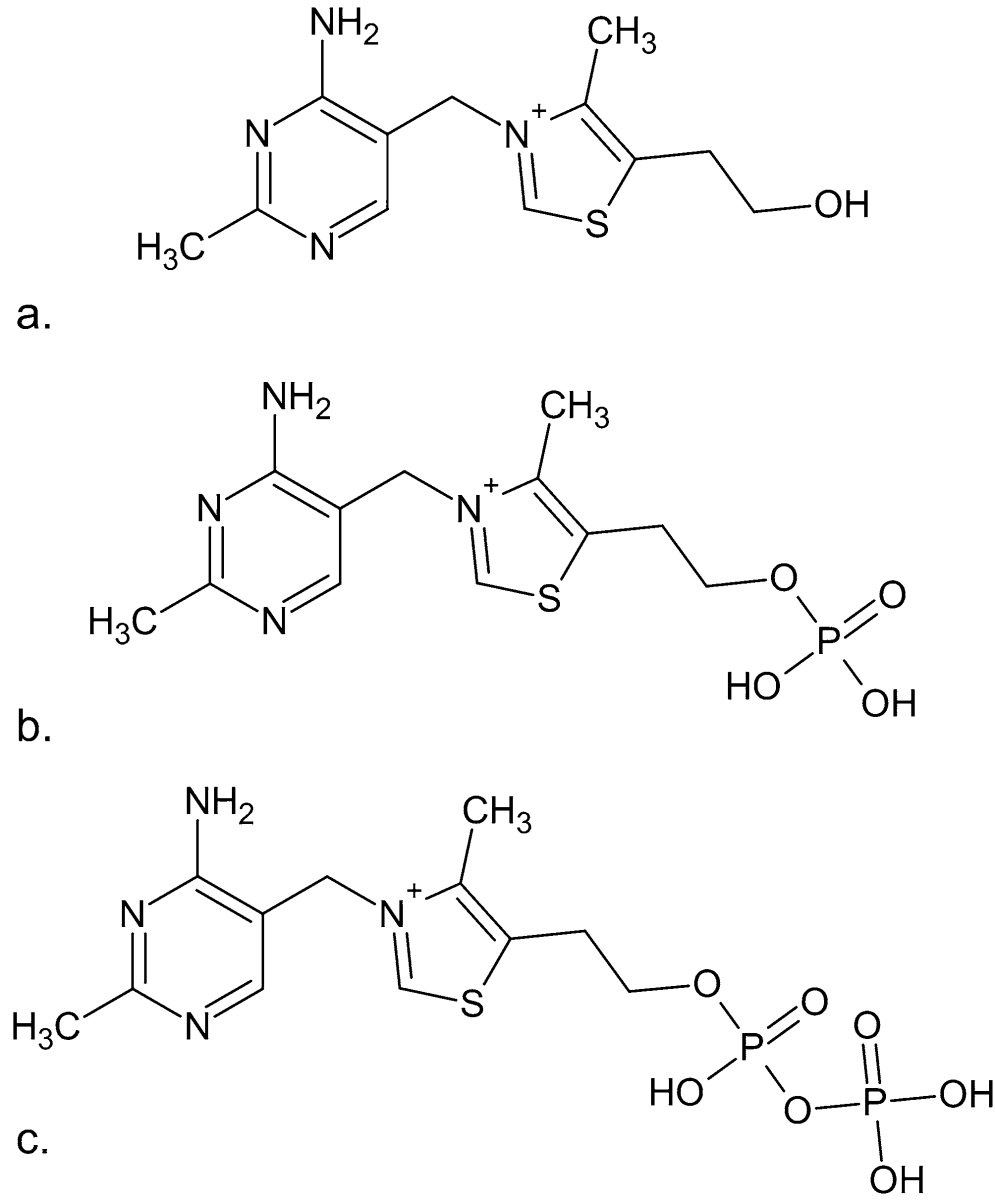
Structures of (**a**) thiamine, (**b**) thiamine monophosphate (TMP), and (**c**) thiamine diphosphate (TDP).

Adsorption is a reversible surface phenomenon resulting from collective non-covalent interactions. Silanol groups in glass can participate in hydrogen bonding and electrostatic interactions with cationic molecules owing to their negative surface charge^16–18^. In contrast, hydrophobic interactions predominate in various synthetic polymeric materials^19^. Thiamine is a highly water-soluble, base-labile small molecule composed of a substituted pyrimidine ring linked to a hydroxyethyl-substituted thiazole ring by a methylene bridge. It is a cation at pH values that are environmentally or physiologically relevant (Fig. 1)^20^.

Cation exchange mechanisms using materials such as Bio-Rex 70 (a macroreticular acrylic polymer matrix with carboxylic acid groups) and Decalso (a synthetic inorganic aluminosilicate)^21^ have been employed for thiamine isolation from foods and human urine^22–27^. Thiamine binding to glass was reported in 1953^28^, and its electrostatic interaction with negatively charged silanol groups has provided the basis for separation on silica-based column packings^29^. Although this adsorption is not a new or unexplainable phenomenon, investigations reporting results from thiamine analysis from environmental samples seldom provide details regarding the composition of collection containers, filtration housing, filtersand vials used in preparing standard solutions and samples. In our examination of potential thiamine losses, we found unexpectedly large losses to glass HPLC autosampler vials and glass fiber filters commonly used in environmental and clinical analyses. Here we report comparative losses of thiamine to various modern polymeric and glass laboratory storage materials and filters (Table 1), with select comparisons carried out using various holding times, temperatures, pH values, and phosphorylated thiamine forms. These losses could be important to understanding the physical conditions responsible for low ambient concentrations of thiamine and associated compounds in environmental samples, as well as how organisms respond to the availability of these compounds.

**Table 1:** Materials investigated for thiamine adsorption.

	Composition
Containers	Type I borosilicate glass tubes; polypropylene, amber glass, silanized and non-silanized glass autosampler vials; polystyrene and polypropylene centrifuge tubes
Filtration apparatus	Glass housing with glass frit and glass filtration flask; polysulfone housing with polypropylene filter support and polypropylene filter flask
Filters, 47 mm	Glass fiber (GF/F (0.7 μm pore size), GF/C (1.2 μm pore size), 0.22 μm polyethersulfone (PES), 0.2 and 0.45 μm cellulose nitrate, 0.2 μm and 0.45 μm cellulose acetate, 0.2 μm nylon, 0.22 μm polycarbonate (PC), 0.2 μm polyamide

## Materials and methods

HPLC autosampler vials were purchased from Waters (LCGC-certified amber glass, 8 × 40 mm), ThermoFisher (polypropylene 8 × 40 mm), and National Scientific (non-silanized and silanized, 1.5 mL vials). 15 mL polypropylene and polystyrene centrifuge tubes were manufactured by Becton Dickinson and Sarstedt, respectively. The 10 × 75 and 12 × 75 mm borosilicate glass tubes were branded as and purchased from VWR. All glass and plastic tubes were new and not previously used. The following 47 mm filters were purchased from Whatman: GF/F, GF/C, 0.2 μm nylon, cellulose acetate, cellulose nitrate, and polyamide; 0.45 μm cellulose acetate; and cellulose nitrate and those from Millipore were 0.22 μm polyethersulfone (PES) and 0.4 μm polycarbonate (Isopore HTTP). HPLC grade water, thiamine hydrochloride, trichloroacetic acid (TCA), sodium hydroxide, and potassium ferricyanide were purchased from VWR or Fisher Scientific. All chemicals were ACS grade. The polypropylene filter flask was manufactured by Nalgene, and the polysulfone filter housing with integrated polypropylene filter support was manufactured by Advantec.

### Storage conditions in various laboratory containers

Thiamine (100 nM) was prepared in deionized tap water and stored as a 1 L volume in a 1 L Pyrex media bottle before transfer to the listed containers as follows: 1 mL of 100 nM thiamine was stored under static conditions in the dark for 1 h at 4 °C and 21 °C in HPLC autosampler vials (8 × 40 mm amber glass or polypropylene, 1.5 mL silanized and non-silanized glass), 15 mL centrifuge tubes (polystyrene and polypropylene), a 125 mL high-density polyethylene (HDPE) bottle, and 10 × 75 and 12 × 75 mm Type I borosilicate glass tubes. 3 × 50 µL of the solutions were transferred to a black microtiter plate and converted to thiochrome by the addition of 100 µL alkaline potassium ferricyanide (75 μL 1% (w/v) potassium ferricyanide diluted to 10 mL with 15% (w/v) NaOH). Standards were prepared by making twofold dilutions of thiamine in HPLC grade water, then adding 100 µL alkaline ferricyanide to 50 µL of standard in triplicate in the same black microtiter plate. Fluorescence detection was carried out at λ_ex_ = 360/40 nm, λ_em_ = 450/50 nm using a FLX800 fluorescence microplate reader (BioTek Instruments, Winooski, VT or at 360/9 nm, λ_em_ = 450/15 nm using a SpectraMax i3x fluorescence plate reader (Molecular Devices, San Jose, CA), as specified in the figure captions. Two different plate readers, at separate sites, were used in these experiments. All fluorescence intensity values were equated to thiamine concentrations using calibration curves on each plate. A similar experiment was repeated on a subsequent date using 100 nM thiamine, thiamine monophosphate (TMP), and thiamine diphosphate (TDP) as separate solutions in HPLC grade water and stored in a 50 mL BD Falcon tube prior to transfer to the same listed containers for 3 h at 21 °C.

### Static storage versus storage with mixing

300 to 750 µL of 100 nM thiamine were stored under static or vortexed conditions for 1 h at 21 °C in 10 × 75 mm and 12 × 75 mm Type I borosilicate glass tubes and 1.5 mL polypropylene and 5 mL polystyrene tubes. The tubes were vortexed moderately every 10 min for 10 s. Results are presented after conversion of the solution to thiochrome using alkaline ferricyanide as described above. The height of the fluid in contact with the tube walls was measured while standing and vortexing. Identical experiments (without the height measurements) were carried out using 100 nM thiamine diluted in 7.5% (w/v) trichloroacetic acid (TCA) and separately 100 nM thiamine converted to thiochrome with alkaline ferricyanide before transfer to storage containers.

### Recovery from filters and filtrates

Thiamine solutions (100 pM to 100 nM in a 125 mL to 1 L volume) were passed through a 47 mm vacuum filtration device with 0.2 to 0.45 μm pore size filters installed. The filters, water type, volumes, and thiamine concentrations were as specified within the description of each trial. The flow rate was dictated by vacuum set at 600 mm Hg. The filters were transferred to 15 mL BD Falcon tubes after no further liquid was observed being removed under vacuum for 2 min, then 2 mL alkaline ferricyanide (0.0075% (w/v) potassium ferricyanide in 15% (w/v) sodium hydroxide) was added to the tubes. The tubes were then vortexed vigorously for 3 min, then centrifuged at 10,000 × *g* for 5 min to settle the filter itself or any particulates formed in the process. 3 × 100 µL of the supernatant was transferred to a black microtiter plate containing 50 µL HPLC grade water. 3 × 50 μL of the initial solutions, filtrates, and thiamine standards were transferred to the same plate, to which 100 μL of alkaline ferricyanide was added. The volume ratio of alkaline ferricyanide (100 µL) to water (50 µL) was the same for the filter supernatants, filtrates and standards for consistency. To determine total pmol thiamine bound, the concentration of the filter sample was multiplied by 20 (2000 μL total volume of alkaline ferricyanide added divided by the 100 µL sampled).

### HPLC analysis

1 mL of a 100 nM equimolar mixture of thiamine, TMP, and TDP in HPLC grade water was stored under static conditions in the dark for 1 h at 21 °C in HPLC autosampler vials (amber, silanized, non-silanized glass, and polypropylene). 874 µL of the solution was then transferred to a polypropylene 8 × 40 mm autosampler vial. 126 µL of an alkaline potassium ferricyanide solution was added and immediately mixed to convert forms of thiamine to their respective thiochromes. The composition of this solution, and the separation conditions below, were modifications of that reported by Brown et al.^30^ 100 µL of this solution was injected using a Waters 717 autosampler, onto a Hamilton PRP-1, 5 μm 150 mm × 4.1 mm column, with a Waters 486 UV detector set at 372 nm, and Waters 474 fluorescence detector set at λ_ex_ = 372 nm, λ_em_ = 433 nm. A Waters 600 pump and controller mediated the gradient between 25 mM ammonium bicarbonate, pH 8.4 (mobile phase A), and 65% (v/v) 25 mM ammonium bicarbonate, pH 8.4/35% (v/v) dimethylformamide (mobile phase B). The gradient was 3 min mobile phase A, changing to 70% mobile phase B over the next 9 min, then a hold at mobile phase B for 5 min before returning to the original condition over 5 min, followed by a 5-min hold in 100% mobile phase A. The flow rate was 1 mL/min. throughout.

## Results and discussion

Thiamine is a complex structure with potential positive charges on the nitrogen atoms in both its pyrimidine and thiazole moieties (Fig. 1). Adsorption of amine-containing molecules onto glass surfaces and silica-based column packing materials has been observed to result in reduced sensitivity and yield carryover in various analytical methods^18,31,32^. At a larger scale, adsorption of amine-containing molecules onto glass reactors used in drug production and subsequent release has been reported, resulting in impurities in the downstream process^18^.

### Adsorptive losses to containers

**Figure 2:**
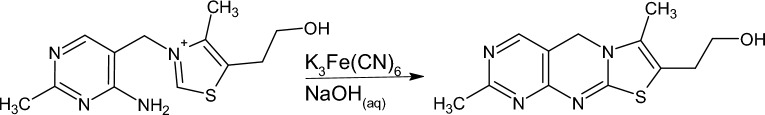
Oxidation of thiamine (non-fluorescent) to thiochrome (fluorescent) using potassium ferricyanide in alkaline solution.

We investigated the recovery of a 100 nM thiamine stock solution following storage at 4 °C and 21 °C in various laboratory vials and tubes, including those made from polypropylene, polystyrene, silanized glass, and non-silanized glass. This concentration was chosen to be relevant to analysis in clinical samples and tissues, plus losses would be within the quantifiable range of standard fluorescence microplate readers. This concentration is approximately 500 to 1000 times higher than in natural environmental water samples; however, such samples routinely undergo pre-concentration steps using solid-phase extraction to bring them into the nanomolar range from commonly observed concentrations ranging from ten to several hundred picomolar^33–35^. In this study, we assessed losses by using the oxidative conversion of the thiamine standard remaining in solution to its fluorescent product thiochrome measured via fluorescence in a microtiter plate format (Fig. 2).

The stock solution of thiamine was stored in a 50 mL polypropylene tube as a control with no apparent losses to this initial container. Additional results, including storage for 1 h in an HDPE bottle, are provided in Fig. S1. In the experiments shown in Fig. S1, we note that the stock solution used as a control was stored as a 1 L solution in a 1 L Pyrex glass media bottle; despite the small surface-area-to-volume ratio of this container, it reduced the initial 100 nM concentration to 88.6 nM.

**Figure 3:**
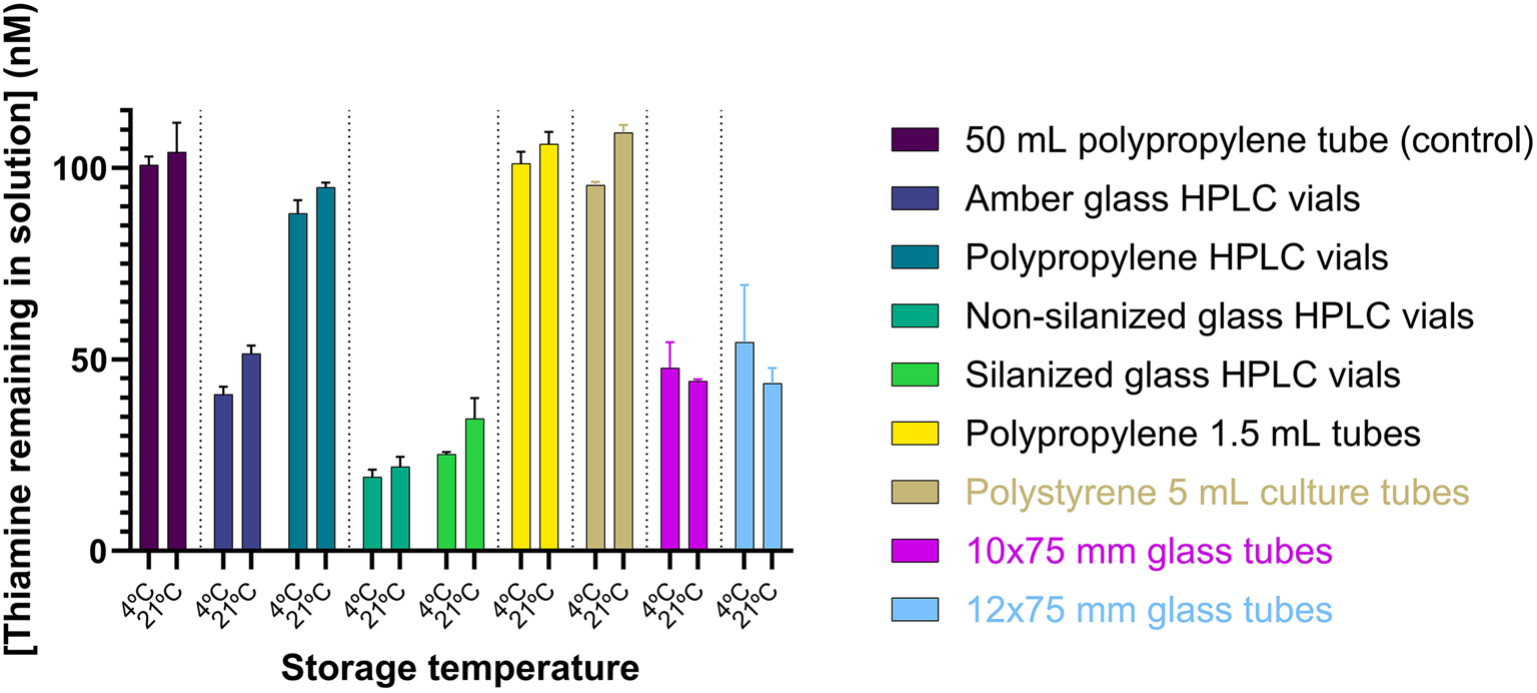
Concentration of thiamine recovered following storage of 1 mL 100 nM thiamine in deionized water for 3 h at 4 °C or 21 °C in 8 × 40 mm 1 mL amber glass and polypropylene autosampler vials, 1.5 mL clear non-silanized and silanized glass autosampler vials, polypropylene 1.5 mL centrifuge tubes, polystyrene 5-mL culture tubes, glass culture tubes (10 × 75 mm) as compared to a 50 mL stock solution stored in a 50-mL polypropylene tube. A vertical dashed line separates materials that may be directly compared based on their surface-to-volume ratios. The results are after conversion of the thiamine remaining in the solution to thiochrome using alkaline ferricyanide with fluorescence detection at λ_ex_ = 360/40 nm, λ_em_ = 450/50 nm.

Given their equivalent surface-to-volume ratios, the materials used in 1 mL 8 × 40 mm amber glass and polypropylene HPLC vials and, separately, silanized versus non-silanized 1.5 mL autosampler vials, were compared directly (Figs. 3, S1). The same comparison was made amongst 15 mL polypropylene and polystyrene centrifuge tubes (Fig. S1). Additional materials were tested (Fig. S1), but due to varying surface-to-volume ratios and limited container types available, a further direct, quantitative comparison between materials was not possible. Our experiments indicated marked losses to all glass containers, most notably non-silanized borosilicate glass HPLC vials, which recovered as little as 19.3 nM of the input thiamine solution at 4 °C (a loss of 80.7 nM, Fig. 3). We similarly saw significant losses of thiamine when diluted in silanized borosilicate glass autosampler vials and Type I borosilicate glass culture tubes. Recovery from silanized glass (25.3 nM recovered) and amber glass (composition not specified, 40.8 nM recovered) HPLC vials was improved, although poor relative to polypropylene HPLC vials (88.1 nM). Silanization, or siliconizing, is a process by which silanol groups (SiOH) in glass are reacted with an alkylsilane to yield siloxanes (Si–O-Si), resulting in increased hydrophobicity and usually reducing the adsorption of polar molecules by blocking electrostatic and nucleophilic interactions^36^. The losses were time-dependent, with more significant losses within the first 30 min of contact time (Fig. S2). The results are consistent with those reported previously, indicating significant losses of thiamine to glassware and the need to pre-treat glassware with alkaline solutions to prevent adsorption^28^.

Limited losses to polypropylene or polystyrene tubes or HDPE bottles were observed. Polypropylene 15-mL conical tubes had a higher thiamine recovery than polystyrene 15-mL tubes (Fig. S1b), and polypropylene showed consistently high recovery across various containers, hence was a preferred storage container material. No clear trends of temperature dependence were noted across materials (Figs. 3, S1).

We then assessed the impact of glass surface area contact by storing solutions of thiamine in 10 × 75 mm and 12 × 75 mm Type I borosilicate glass tubes from the same manufacturer stored either without mixing or with vortexing every 10 min over 1 h. The fluid height in each tube was measured, and the surface area in contact with the fluid was calculated (Table S1). Polypropylene (1.5 mL) and polystyrene (5 mL) tubes were used as material comparators. Losses to the glass tubes maintained statically were greater with smaller tube inner diameter (e.g. − 44.0 nM recovered-versus-94.2 nM recovered for 300 µL stored in 10 mm-vs-12 mm outer diameter tubes, respectively). We note that at 300 µL volumes, the solution was in contact with only the curved hemisphere bottom of the glass tubes for the 12 × 75 mm tubes, whereas this same volume reached the cylindrical sides of the 10 × 75 mm tubes (Fig. S3). Despite a similar surface-area-to-volume ratio, the thiamine losses to the 10 × 75 mm tubes were much greater than the 12 × 75 mm tubes at this volume. As the volumes increased, the fluid in both tube diameters extended further into the cylindrical portion of the tubes, increasing the surface area in contact with the fluid and consequent thiamine losses. A detailed consideration of glass composition and processing is beyond the scope of this study. For further consideration, readers are directed to extensive studies on the properties of glass vials for parenteral formulations and characterization of within lot variability, surface imperfections, and heterogeneities in chemical composition^37–39^. However, the formation of the sealed bottom of test tubes includes direct application of a high-temperature flame and injection of air^40^, which we postulate may yield a material with different adsorption characteristics than the cylindrical sides. Indeed, differences in the chemical composition and the availability of inorganic elements in regions of glass subjected high heat has been demonstrated^37^.

**Figure 4:**
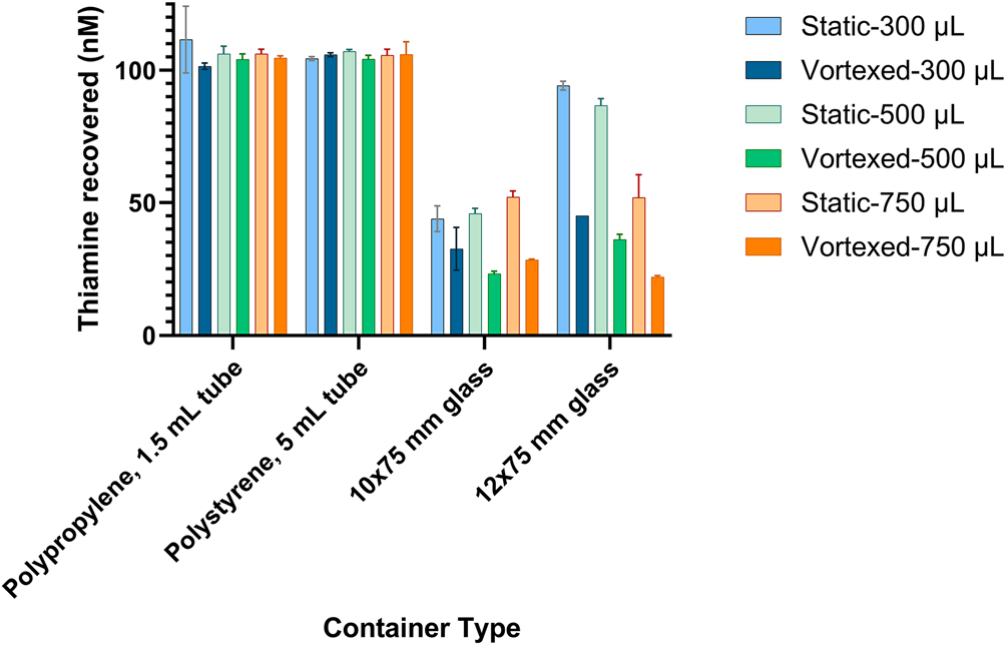
Concentration of thiamine recovered after storage of 100 nM thiamine in HPLC grade water in 10 × 75 mm and 12 × 75 mm Type I borosilicate glass tubes and polypropylene (1.5 mL) and polystyrene (5 mL) tubes under static or vortexed conditions for 1 h at 21 °C. The tubes were vortexed moderately every 10 min for 10 s. The results are after conversion of the thiamine remaining in the solution to thiochrome using alkaline ferricyanide with fluorescence detection at λ_ex_ = 360/40 nm, λ_em_ = 450/50 nm.

Upon vortexing, we observed a significant decrease in free thiamine at all volumes and both glass tube diameters, indicating that adsorptive effects increased with greater contact of the solution to the cylindrical walls of the tube. Periodically vortexing 750 µL of a 100 nM solution of thiamine in a 12 × 75 mm borosilicate glass tube (10 mm inner diameter) allowed for recovery of only 21.9 nM in the solution after 1 h (Fig. 4). The same volume maintained stationary for the same period allowed recovery of 52.0 nM. No thiamine losses to polypropylene or polystyrene were observed under the conditions tested.

An identical experiment with thiamine diluted in 7.5% (w/v) trichloroacetic acid, a commonly used extraction solution for thiamine from tissues, indicated no significant material or vortex-dependent losses (Fig. S4). The protonation of silanol groups at low pH likely blocks electrostatic interactions with thiamine. It is also possible that the positively charged thiamine forms an ion-pairing complex with trichloroacetate^41^, potentially mitigating electrostatic interactions. We further investigated the pH dependence of thiamine adsorption in HPLC-grade water in various glass containers, using pH values below 7.0 to ensure that the stability of alkaline-labile thiamine was maintained. Extensive losses without any pH dependence were observed on non-silanized glass vials (Fig. S5), whereas a slight pH dependence with maximum losses at pH 4.5 was observed in silanized glass vials. The pKa values of silanol groups on quartz, as an exemplar of pure glass, have been reported to be 4.8, 8.5–9.3, and > 11.0^42^. The structure of thiamine undergoes complex changes with pH^43^. The reported pKa values of thiamine are ~ 4.8 on the pyrimidine N1 nitrogen and 9.2–9.3 on the thiazole nitrogen^44,45^. At higher pH, thiamine undergoes ring opening of the thiazole ring to yield the thiol form with a pKa value of 11.6^46^. At the pH values used in this study, some proportion of the more acidic silanol groups would be expected to be negatively charged, while thiamine would be net positively charged at one or both sites. Methylene blue, a cationic dye, is reported to participate in ion-exchange with sodium and hydrogen ions in glass and thus is commonly used to stain glass materials to visualize defects^37,38^. However, the lack of a strong pH dependence to adsorption suggests that electrostatic interactions were not the exclusive mechanism for our observed thiamine losses. In preliminary experiments, thiochrome formed in situ through oxidation of thiamine with alkaline ferricyande exhibited no notable losses to glass, with only minor losses to polystyrene and polypropylene (Fig. S6). Thiochrome lacks the positive charge and increases its hydrophobicity upon ring closure. Losses of aromatic ring containing organic molecules to polystyrene have been attributed to hydrophobicity and π–π interactions^47–49^.

### Effect of containers on thiamine speciation

**Figure 5:**
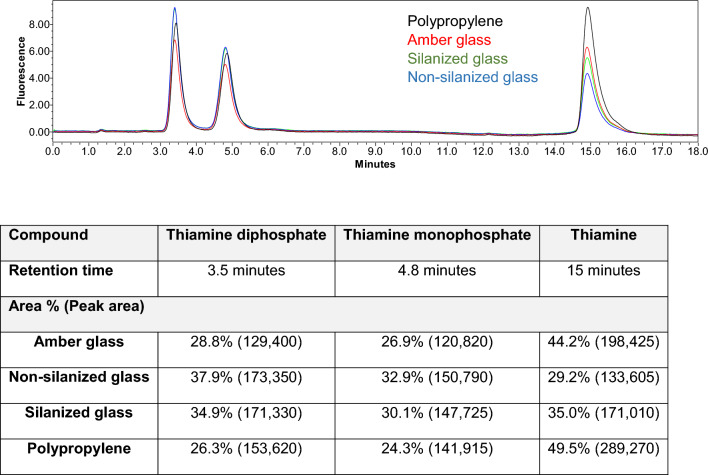
Chromatogram showing recovery of thiamine following storage of 1 mL of a 100 nM equimolar mixture of thiamine, TMP, or TDP stored for 1 h at 21 °C in HPLC grade water in Amber glass (red), clear non-silanized (blue), clear silanized glass (green), and polypropylene (black) HPLC vials prior to removal of the solution and conversion to thiochrome in polypropylene HPLC vials and HPLC analysis with fluorescence detection at 374 nm excitation and 433 nm emission. The peak areas and area % after integration are listed in the table below the figure.

Selected studies in various containers were repeated using thiamine monophosphate (TMP), thiamine diphosphate (TDP), or thiamine alone (Fig. S7), and in a mixture (Fig. 5). Stock solutions stored in polypropylene tubes served as a comparative material control. The parent molecule thiamine is used in dietary supplements and fortification strategies for rice and grains. TDP is the active cofactor form of thiamine for key metabolic enzymes, including transketolase in the pentose-phosphate pathway, pyruvate dehydrogenase linking glycolysis to the citric acid cycle, and branched chain α-keto acid dehydrogenase and α-ketoglutarate dehydrogenase in the latter. The availability of the phosphorylated forms relative to the parent molecule provides insight into dietary intake, systemic absorption, and the efficiency of conversion of thiamine to TDP by thiamine pyrophosphokinase, which various conditions can impede^30,50^.

Consistent with prior results, thiamine was largely not lost to polypropylene or polystyrene tubes (Fig. S7). This paralleled the findings with TMP and, to a significant extent, TDP. Notably, the substantial observed thiamine losses to glass containers were not observed with TMP or TDP. TMP and TDP could maintain the cation on the thiazole nitrogen and pyrimidine N1 nitrogen but contribute negative charges due to the attached phosphate groups that can be increasingly deprotonated with increasing pH (Fig. 1). Thus, diminished loss of these phosphorylated compounds could be attributed to reduced likelihood of electrostatic interactions. Still, amber glass vials exhibited moderate losses of TMP and TDP (75–80% recovery).

Likewise, selective losses were seen when the thiamine forms were combined in an equimolar mixture. Assuming no losses to polypropylene tubes when thiamine comprised 49.5% of the chromatogram peak area (the condition used as a control, peak area 289,270), a marked reduction in the peak area (most notably, with non-silanized glass, 29.2%, peak area 133,605) was observed selectively for thiamine stored in all forms of glass (Fig. 5). This equated to a loss of 53.8% of thiamine peak area and indicated a greater loss of thiamine relative to phosphorylated forms when stored in glass, which could skew the interpretation of sample data and complicate comparisons between published studies. The loss of TMP and TDP to amber glass observed for individual compounds was maintained in this mixture, with a 14.9% and 15.8% reduction in peak areas, respectively, relative to storage in polypropylene autosampler vials. This suggested that a mechanism beyond electrostatic interactions could be present as the losses to amber glass carried over to the phosphorylated derivatives.

Amber glass is commonly made by adding metal oxides such as those from iron and manganese to impart protection from UV light^51^. A previous study found greater breakdown of amitryptiline (a tricyclic antidepressant) when stored in amber glass ampules than in clear glass ampules, attributed to a free-radical oxidation process accelerated by the presence of metal ions^52^. These authors noted the increased concentration of iron in the former and found measurable concentrations of extractable iron at low pH and elevated temperature (pH 3 and 80 °C.) A study with naloxone, nalbuphine, and oxymorphone similarly found higher amounts of their oxidation products after storage in amber glass vials versus clear vials, noting the presence of 0.3 ppm iron in solutions stored in amber glass vials at ambient temperature for 4 h, but undetectable levels with clear vials^53^. As we assayed the concentration of thiamine remaining in solution after storage by measuring thiochrome fluorescence, we cannot exclude the possibility of thiamine degradation by metal ion leachates from the glass containers and formation of non-fluorescent products. Thiamine has been shown to exhibit decomposition upon storage in the presence of iron, magnesium and copper compounds^54–56^, and trace metals in glass could potentially contribute to thiamine losses.

### Losses to filtration devices and filters

We also tested the impact of various filter materials and filtration apparatuses. We observed thiamine loss from solutions containing 100 nM thiamine when passed through a standard laboratory 1 L glass filtration vacuum apparatus where the filter housing, integrated frit, and collection flask were glass (Fig. S8). Despite limited contact time (20 s, Table S2), significant thiamine losses from solutions passed through the glass filtration apparatus alone were observed (loss of 10.2 to 12.2 nM) relative to the control stored separately in a polypropylene container (Fig. S9). We subsequently employed and recommend using a filter apparatus where the filter housing, integrated frit, and collection flask are plastic (polysulfone filter housing, polycarbonate filter support, and polypropylene filter flask, Fig. S8). Using this filter device, the losses of thiamine under the same conditions were reduced to 3.6 nM.

**Figure 6:**
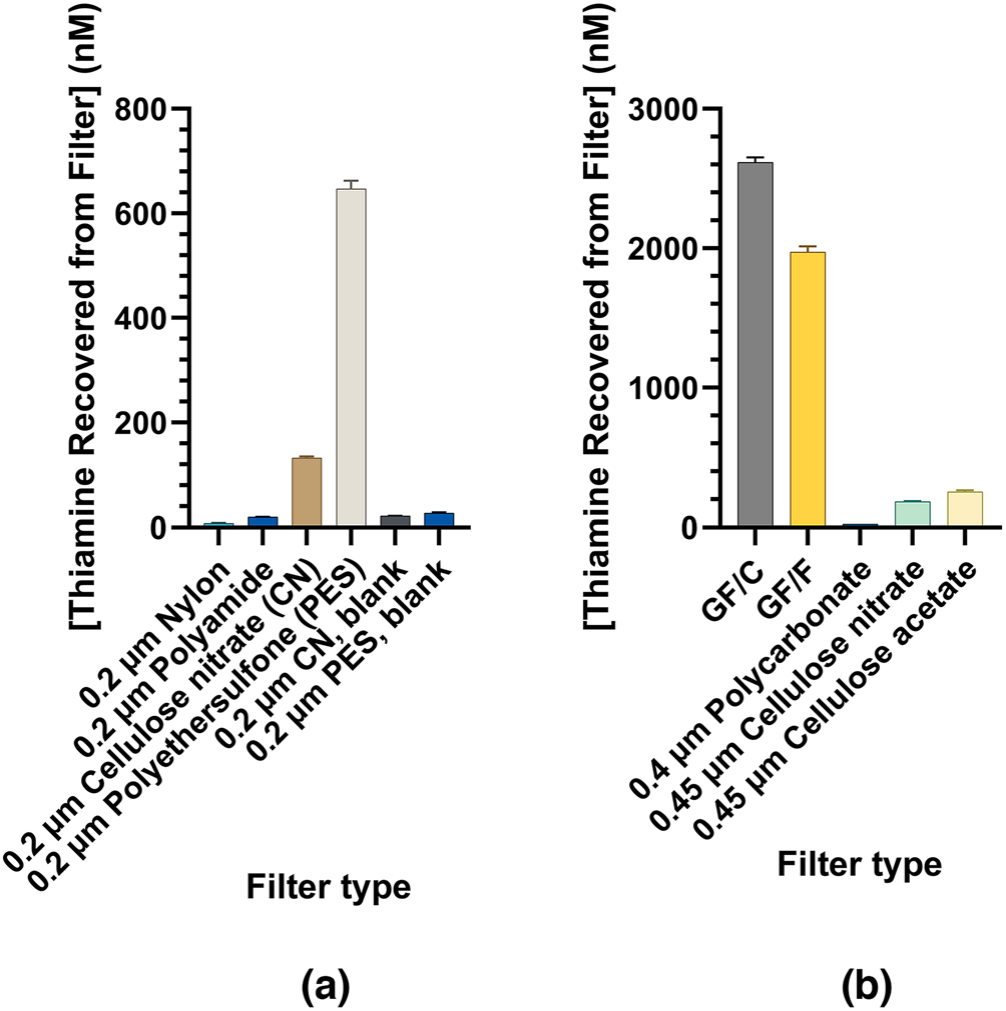
Filter retention of 100 nM thiamine in deionized water following passage of (**a**) 200 mL through 0.2 µm 47 mm polyamide, cellulose nitrate (CN), polyethersulfone (PES), and nylon filters. Water only containing no thiamine was used as a negative control through CN and PES membranes. (**b**) 200 mL through glass fiber (GF/F and GF/C), and 0.4 μm polycarbonate, 0.45 μm CN, and 0.45 μm cellulose acetate membranes. The results are after vortexing filters in 2 mL alkaline potassium ferricyanide to convert filter-retained thiamine to thiochrome followed by fluorescence detection at λ_ex_ = 360/40 nm, λ_em_ = 450/50 nm. Error bars represent one standard deviation of triplicate thiochrome determinations of the membrane extract.

To assess the extent of thiamine losses from solution and adsorption on filters, we passed 100 to 1000 mL of water spiked with 100 pM to 100 nM thiamine through 47 mm membrane filters of varying compositions. The filters were vortexed in 2 mL alkaline ferricyanide for 2 min to convert adsorbed thiamine to thiochrome, centrifuged, and the supernatant was assayed by fluorescence. We previously used alkaline ferricyanide to simultaneously release thiamine from an immobilized binding protein and convert it to fluorescent thiochrome^57^, hence, the same strategy was applied for release from filter matrices. We assayed the thiamine recovered in the filtrates and the thiamine remaining bound to the filters following conversion to thiochrome. The results for the filters themselves are shown in Fig. 6, with the corresponding filtrates in Fig. S9. Tens to hundreds of picomoles of thiamine were recovered from the filters through extraction with 2 mL of alkaline ferricyanide, leading to a 50 to 500-fold concentration factor depending on the initial volume (100 to 1000 mL). This selective binding and volume reduction permitted the detection of standards in the micromolar range that were initially in the nanomolar range.

Of the 47 mm filter materials tested (Table 1), the greatest recovery of thiamine using alkaline ferricyanide was found in the filtrate from filtration through 0.2 μm nylon, 0.2 μm polyethersulfone (PES), and 0.45 μm cellulose acetate. Filtrates from other membranes, most notably glass fiber (0.7 and 1.2 μm) and cellulose nitrate (0.2 and 0.45 μm), resulted in significant recovery losses in the filtrate relative to the processed control (Fig. S9, 125 mL of 100 nM thiamine concentration passed through the filtration apparatus only). Losses in the filtrate to filtration from PES, cellulose acetate, and polycarbonate membranes were moderate (3.9 nM, 3.7 nM, and 7.7 nM, respectively, relative to the processed control). However, the losses to the filtrate from filtration through cellulose nitrate and borosilicate glass fiber filters were substantial, equating to losses of as much as 24.8 nM and 30.3 nM, respectively. We note that in this material screening, we maintained a constant vacuum, but this resulted in material-dependent and pore-size dependent flow rates (Table S2). Decreased flow rates would increase the contact time and potential for non-specific binding and losses of thiamine in the filtrate. Solely looking at flow rates, we would expect the greatest losses to the filters from nylon, which was in contact with the thiamine solution for the longest period (5 min, 42 s), however, glass fiber filters which were in contact for the least amount of time (30–50 s) exhibited the highest losses from the solution.

Of the filters themselves, glass fiber, PES, and cellulose nitrate filters yielded detectable thiamine retention. 2.7 μM (Fig. 6b) thiamine was recovered from the GF/C filter, indicating significant retention by this glass fiber filter. Moderate retention was observed by PES and cellulose nitrate filters (Fig. 6a, 647 nM and 132 nM, respectively). It is important to highlight that the basis for the up to low μM filter recoveries from the initial 100 nM thiamine solution stems from both the filter material-dependent binding of thiamine as well as 50 to 500-fold lower volume used to extract the filters. For example, the 2.7 μM thiamine recovered from the GF/C glass fiber membranes corresponds to 5.4 nmol thiamine in the 2 mL alkaline ferricyanide extraction volume from an initial 200 mL of a 100 nM solution (20 nmol). We suggest nylon, cellulose acetate, or polycarbonate membranes for routine thiamine analysis of filtrates, as all yielded high recoveries in the filtrates and relatively minor retention on the filters. However, we note that we did not conduct further work with these membrane materials, and further characterization for pH dependence and capacity may be warranted.

For filter materials exhibiting notable thiamine retention (glass fiber, cellulose nitrate, and polyethersulfone), we assessed possible signals from the filters themselves in the absence of thiamine when treated identically with alkaline ferricyanide (Fig. S10a). We observed the highest autofluorescence from the extract of PES, followed by cellulose nitrate, then at a much lower intensity, from glass fiber membranes, all of which were significant relative to unfiltered water. The autofluorescence from cellulose nitrate was not surprising as it has been noted to be a factor in signal determination from microarrays, Western blots, and lateral flow assays^58,59^. However, this autofluorescent signal provided a negligible background to the fluorescence signal obtained from membranes in the presence of thiamine, indicating that the signal from the filters was due to the presence of thiamine rather than a non-specific signal from the membranes (Fig. S10b). The composition of the material was the primary influence upon this binding, with binding to glass fiber membranes substantially higher than other materials despite a faster flow rate (Table S2) and comparatively large pore sizes.

The high binding exhibited by some materials in our study to understand adsorptive losses prompted in parallel an assessment of whether we could leverage the binding by filter materials to pre-concentrate thiamine to simplify its analysis in dilute solutions. Glass fiber and PES membranes were studied further for pH dependence and capacity as these materials showed notable binding. The results from these experiments are discussed below.

### Potential impact on the analysis of environmental samples

As thiamine is present at low levels in the environment (low pM range in lake and water samples) as well as in plasma and tissue samples (low nM range)^1–5^, even minor losses to collection vessels, storage vessels, filters, and filtering apparatuses need to be considered when interpreting results. Filters made of various materials, including nylon, PTFE, PES, and polypropylene, have been reported as used for particulate removal during thiamine sample preparation in a variety of studies^33–35,60–63^. Following filtration and prior to storage at − 20 °C, environmental water samples have been collected in borosilicate glass bottles^64^, acid-washed plastic bottles, including polyethylene^33–35,63,65^ or in containers with unspecified polymer composition^2^, though the composition of the filtration apparatus and autosampler vials usually is not detailed.

**Figure 7:**
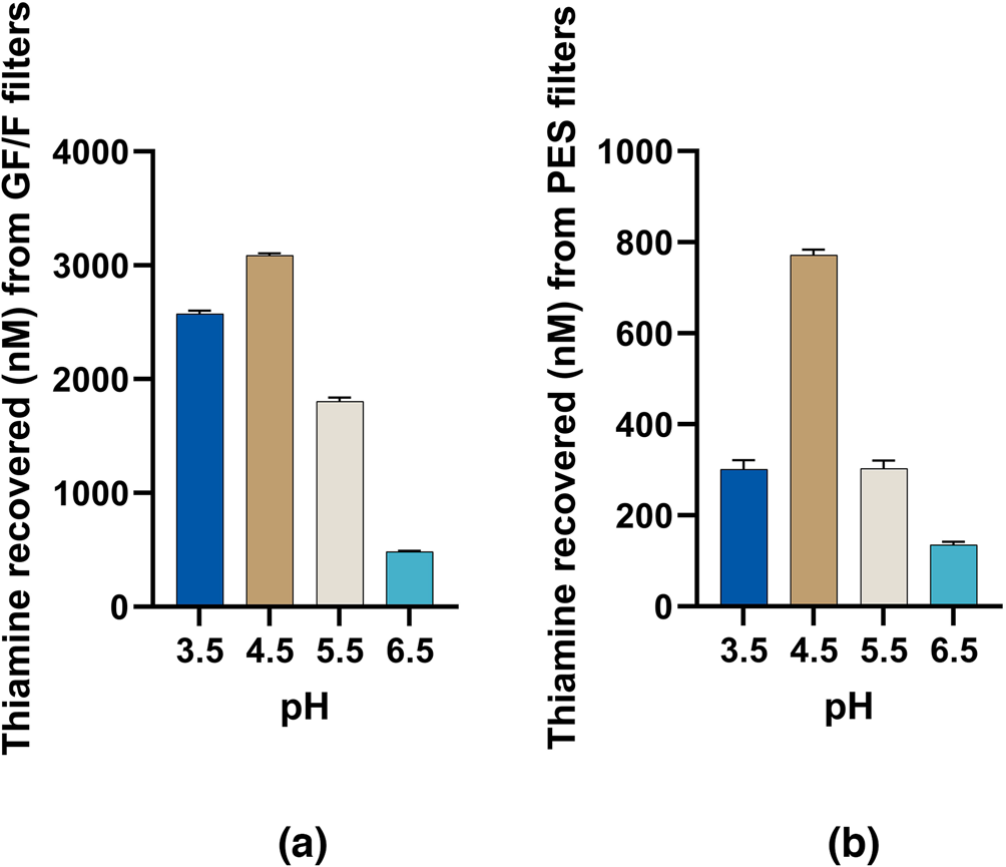
Concentration of thiamine recovered following filtration of 125 mL 100 nM thiamine in commercially bottled HPLC grade water pH adjusted to 3.5–6.5 through (**a**) GF/F glass fiber (0.7 μm pore size) and (**b**) 0.2 µm polyethersulfone (PES) filters after vortexing filters in 2 mL alkaline potassium ferricyanide. The results are after conversion of the filter-retained thiamine to thiochrome using alkaline ferricyanide with fluorescence detection at (**a**) λ_ex_ = 360/40 nm, λ_em_ = 450/50 nm and (**b**) λ_ex_ = 360/9 nm, λ_em_ = 450/15 nm.

Given the extensive binding of thiamine on glass fiber and, more modestly, PES, we sought to understand the impact of these materials on thiamine recovery. Further, given the efficiency of binding and ease of recovery of thiamine’s fluorescent oxidation product, we considered whether the losses to these filter materials could be leveraged for thiamine isolation to simplify downstream analyses. Currently reported methods for quantification of thiamine in environmental water rely on filtration through 0.2 μm pore size membranes, pH adjustment to 6.5, followed by solid-phase extraction using C18 cartridges^33–35^. To maximize binding, we examined the pH dependence of thiamine adsorption on PES and glass fiber filters as examples of moderate and high-binding materials, respectively (Fig. 7). Adsorption to both filter materials was maximal at pH 4.5, consistent with the results obtained in silanized glass autosampler vials. However, the pH dependence of the filters was more distinct. PES is reported to have a negative charge at pH values tested within and a decreasing zeta potential with increasing pH^66,67^.

Prior efforts relying on cation exchange for thiamine analysis on Bio-Rex 70 or Decalso investigated factors including ion exchange material amount, sample flow rate, eluent composition, temperature, and volume^22,27^. In our experiments, the filter diameter was fixed, and the elution volume in alkaline ferricyanide was intentionally minimized to maximize the concentration factor. We varied the sample application flow rate, finding ~ 33% greater recovery with a 20 min/L flow rate versus a 5 min/L flow rate, indicating that retention was improved on glass fiber filters with greater contact time (Fig. S11).

**Figure 8:**
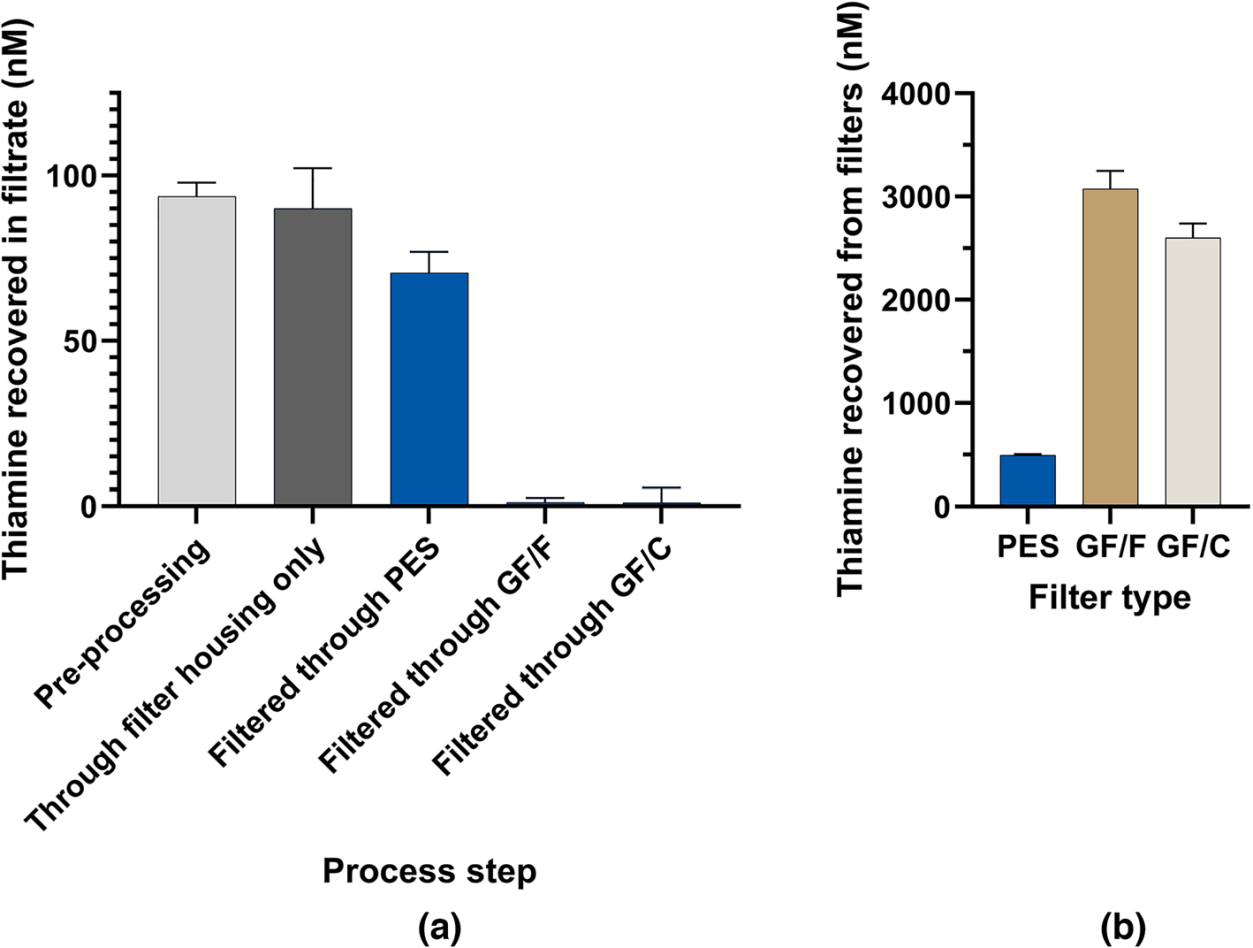
Recovery of thiamine following filtration of 125 mL 100 nM thiamine in HPLC grade water pH adjusted to 4.5 through 0.2 µm polyethersulfone (PES), GF/F glass fiber (0.7 μm pore size) and GF/C glass fiber (1.2 μm pore size) membranes relative to the pre-processed sample and that passed through the plastic filter housing and flask only. (**a**) The concentration of thiamine recovered in the filtrate. (**b**) Concentration of thiamine recovered from the filters after vortexing filters in 2 mL alkaline potassium ferricyanide. The results are after conversion of (**a**) thiamine remaining in solution and (**b**) the filter-retained thiamine to thiochrome using alkaline ferricyanide with fluorescence detection at λ_ex_ = 360/9 nm, λ_em_ = 450/15 nm.

We passed 125 mL of a 100 nM solution of thiamine diluted in commercially bottled HPLC grade water adjusted to pH 4.5 through GF/F, GF/C, and PES filters (Fig. 8). Notably, only 1 nM of the original 100 nM thiamine stock was detected in the filtrate following passage through either GF/F or GF/C filters, relative to 75 nM with PES filters (Fig. 6a). GF/C and GF/F borosilicate glass filters are specified as having 1.2 and 0.7 µm pore sizes, respectively, while the PES membrane had a pore size specification of 0.2 μm. Thiamine recovery from the GF/C and GF/F 47 mm filters was 2.7 μM and 3.1 µM, respectively owing to the membrane retention and the significantly lower elution volume, compared to 0.49 μM on PES (Fig. 8b). In these experiments, a total of 12.5 nmol of thiamine was passed through the membranes, and as much as 6.2 nmol (49.6%, 3.1 μM in the 2 mL extraction volume) was recovered on the GF/F filter. The losses of thiamine to the filters may have been higher than our results indicate, since we did not obtain quantitative recovery of thiamine between filtrates and filter extracts. It is known that oxidative conversion of thiamine to thiochrome is a competitive process with the formation of non-fluorescent products^20^ and we cannot exclude the potential contribution of the filter material in this conversion. While the analysis of thiamine remaining in the filtrates from a solution of known concentration is straightforward, the efficiency of the extraction of the filters with alkaline ferricyanide and simultaneous conversion to thiochrome may occur in a filter material-dependent manner.

**Figure 9:**
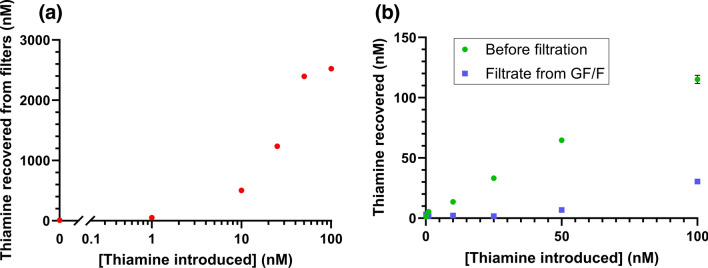
Recovery of thiamine following filtration of 125 mL 0–100 nM thiamine in HPLC grade water pH adjusted to 4.5 through 47 mm 0.7 µm pore size glass fiber (GF/F) membranes. (**a**) The concentration of thiamine recovered from the glass fiber filters after conversion to thiochrome in 2 mL alkaline ferricyanide. (**b**) The concentrations of the filtered solutions were determined before (green circles) and after (blue squares) filtration. The results are after conversion of (**a**) the filter-retained thiamine and (**b**) thiamine remaining in solution to thiochrome using alkaline ferricyanide with fluorescence detection at λ_ex_ = 360/40 nm, λ_em_ = 450/50 nm. Each point is the average of triplicate wells used for thiochrome fluorescence with error bars representing their standard deviation.

These experiments were repeated over a range of thiamine concentrations for GF/F and PES filters. For GF/F filters, we observed an approximately 50-fold concentration factor, nearing apparent saturation of the filter binding capacity at 100 nM (Fig. 9a). The thiamine concentration remaining in the filtrate was markedly reduced at all introduced concentrations, with notably only 5% of the thiamine from the 25 nM solution detected in the filtrate (Fig. 9b). For PES filters, we found an approximately 12-fold concentration factor and linear response through 100 nM (Fig. S12).

Upon further experimentation, we found that PES exhibited notable binding in some trials and less so in others, which we can only attribute to possible differences in the source water (house deionized water). When applied to environmental water samples, our results indicated that thiamine could still be retained on GF/F filters despite a substantially more complicated matrix. River water (250 mL) spiked with 10 nM thiamine yielded fluorescence at thiochrome wavelengths that was discernable from the background, but following filtration through GF/F filters, this signal was lost (Fig. S13a). When analyzing the filters themselves, an apparent recovery of 180 nM (Fig. S13b) was noted relative to 100 nM in the water without the spike. This suggested that even this relatively high thiamine concentration could be lost to filtration in a complex matrix. We considered whether using GF/F filters may have value in cleaning up natural water samples for thiamine analyses. However, the high background fluorescence in natural matrices unrelated to thiamine, the expected variation in flow rates, and the variable competitive effects likely from other matrix constituents, such as Ca^2+^ or Mg^2+^ ions or other organics, precluded further investigation at this time. In addition, preliminary screenings of 1 L lake, creek, and tap well waters, unspiked and spiked with 100 pM thiamine (a realistic concentration in environmental water), and filtered through GF/F filters did not bring these samples to a detectable range. This did not exclude thiamine retention onto the fibers, but thiamine potentially retained and recovered was below the instrumental limit of detection (8 nM). In addition, we cannot exclude the likelihood of thiamine sorption to particulates^68^ smaller than the 0.7 μm membrane pores that would have been lost to the filtrate.

We cannot generalize the impact of adsorption in published methods due to variations in sample matrices, thiamine concentrations, pH values, and contact with filters, collection and storage containers of varied or unspecified materials or sources. Thus, we recommend that the impact of adsorptive losses should be determined empirically for any given analytical process. Fortunately, the commonly applied method of thiamine extraction from tissues in trichloroacetic acid is unlikely to result in significant adsorption losses of thiamine (Fig. S4). Similarly, thiochrome formed in situ from thiamine was not notably affected by the storage container composition in preliminary experiments (Fig. S6). Thus, depending on the requirement of the downstream analysis, we suggest that thiamine samples should be stored in polypropylene or polystyrene tubes, in trichloroacetic acid, or first converted to thiochrome. Polypropylene exhibited no detectable thiamine adsorption but has additionally been reported to be resistant to 100% trichloroacetic acid at 60 °C for several months^69^. Thiamine adsorption is a more substantial concern when the samples are collected in or introduced following a purification procedure such as SPE and analyzed in their native form by LC-UV, LC–MS, or LC-MS^2^ or with a post-column derivatization procedure to generate thiochrome in situ following chromatographic separation. In future work, we plan to follow up on the losses of thiamine, TMP and TDP to amber glass vials by LC–MS to characterize their potential to form oxidized products that are not detectable by routinely used fluorescence-based assays.

Aside from glass^28^, thiamine adsorption has been investigated in plastic particles of various compositions used in food packaging^70^, onto clays and soils^68^, and recognition of thiamine by charge-based interactions to various nanoparticles in the absence of a specific biorecognition event forms the basis of numerous reported biosensing technologies^20^. When adsorption experiments are carried out on particles, it is critical to consider the container and diluent used in the experiments and imperative to include a thiamine standard processed identically at all steps to account for losses that are independent of the particles. When sensors based on non-covalent interactions are developed in the absence of a specific biorecognition event, it is critical to examine the specificity of these interactions in relevant complex matrices. In microbial growth experiments where the knowledge of the thiamine concentration is central to the investigation, it is important to validate the washing protocol to avoid carryover^71,72^. For this purpose, Sannino et al. developed an extensive washing protocol for glassware involving a commercial cleaning solution, extensive rinsing with water, baking overnight at 200 °C, washing in 0.1 N sodium hydroxide, followed by water rinsing and baking again^71^. In future work, we plan to determine how impactful adsorption effects are on bacteria cultured under thiamine-limited growth conditions in common culture tubes and media bottles.

One universal concern raised by our findings is the accuracy of calibration curves prepared in a clean matrix (e.g., HPLC-grade, distilled, or deionized water) relative to the samples. If thiamine standards are prepared in purified water either in glassware or subsequently stored in glass autosampler vials, adsorptive losses to the containers would result in a lower signal for a given input concentration of thiamine. If the samples analyzed using this calibration curve for comparison were in a matrix that prevented significant thiamine adsorption, the sample thiamine content could be overestimated. Further, in experiments where speciation of thiamine forms is essential, the selective loss of thiamine, relative to TMP and TDP, to glass containers would overestimate the proportion of the latter compounds relative to the parent molecule. In future work, we plan to carry out similar experiments of other vitamins and biologically relevant phosphorylated small molecule analytes to understand the universality of our findings.

### Supplementary Information

Supplementary Information.

## Data Availability

The datasets supporting the conclusions of this article are available upon reasonable request from the corresponding author.

## References

[1] Ohwada K, Taga N (1972). Vitamin B12, thiamine, and biotin in Lake Sagami. Limnol Oceanogr..

[2] Natarajan KV (1968). Distribution of thiamine, biotin, and niacin in the sea. Appl. Microbiol..

[3] Carlucci AF, Bowed PM (1972). Determination of vitamin B12, thiamine, and biotin in Lake Tahoe waters using modified marine bioassay techniques. Limnol. Oceanogr..

[4] Kurata A, Kadota H (1981). Annual changes of vitamin B1, biotin and vitamin B12 in water in Lake Biwa. J. Nutr. Sci. Vitaminol. (Tokyo).

[5] Moskowitz A, Graver A, Giberson T (2014). The relationship between lactate and thiamine levels in patients with diabetic ketoacidosis. J. Crit. Care.

[6] Kraft CE, Angert ER (2017). Competition for vitamin B1 (thiamin) structures numerous ecological interactions. Q. Rev. Biol..

[7] Balk L, Hägerroth P, Gustavsson H (2016). Widespread episodic thiamine deficiency in Northern Hemisphere wildlife. Sci. Rep..

[8] Fitzsimons JD, Williston B, Zajicek JL (2005). Thiamine content and thiaminase activity of ten freshwater stocks and one marine stock of alewives. J. Aquat. Anim. Health.

[9] Honeyfield D, Hinterkopf J, Fitzsimons J, Tillitt D, Zajicek J, Brown S (2005). Development of thiamine deficiencies and early mortality syndrome in lake trout by feeding experimental and feral fish diets containing thiaminase. J. Aquat. Anim. Health.

[10] Rowland FE, Richter CA, Tillitt DE, Walters DM (2023). Evolutionary and ecological correlates of thiaminase in fishes. Sci. Rep..

[11] Tomasulo PA, Kater RM, Iber FL (1968). Impairment of thiamine absorption in alcoholism. Am. J. Clin. Nutr..

[12] Davis RE, Icke GC (1983). Clinical chemistry of thiamin. Adv. Clin. Chem..

[13] Kritikos G, Parr JM, Verbrugghe A (2017). The role of thiamine and effects of deficiency in dogs and cats. Vet. Sci..

[14] Shamir R (2012). Thiamine-deficient infant formula: What happened and what have we learned?. Ann. Nutr. Metab..

[15] Chang Y-P, Chiu P-Y, Lin C-T, Liu I-H, Liu C-H (2017). Outbreak of thiamine deficiency in cats associated with the feeding of defective dry food. J. Feline Med. Surg..

[16] Behrens SH, Grier DG (2001). The charge of glass and silica surfaces. J. Chem. Phys..

[17] Ngo D, Liu H, Chen Z (2020). Hydrogen bonding interactions of H_2_O and SiOH on a boroaluminosilicate glass corroded in aqueous solution. NPJ Mater. Degrad..

[18] Xu X, Lott J, Kelly KA, Shi Z (2022). Adsorption of amine compounds on the glass surface and their impact on the development of analytical method and pharmaceutical process. Org. Process. Res. Dev..

[19] Illum L, Bundgaard H (1982). Sorption of drugs by plastic infusion bags. Int. J. Pharm..

[20] Edwards KA, Tu-Maung N, Cheng K, Wang B, Baeumner AJ, Kraft CE (2017). Thiamine assays—Advances, challenges, and caveats. ChemistryOpen..

[21] Nachod FC, Wood W (1944). The reaction velocity of ion exchange. JACS..

[22] Pippen EL, Potter AL (1975). Quantitating the recovery of thiamine (vitamin B1) from Decalso in the thiochrome method for the determination of thiamine. J. Agric. Food Chem..

[23] Jowett M (1940). The estimation of vitamin B(1) in urine. Biochem. J..

[24] Roser RL, Andrist AH, Harrington WH, Naito HK, Lonsdale D (1978). Determination of urinary thiamine by high-pressure liquid chromatography utilizing the thiochrome fluorescent method. J. Chromatogr. B Biomed. Sci..

[25] Zajicek JL, Tillitt DE, Honeyfield DC, Brown SB, Fitzsimons JD (2005). A method for measuring total thiaminase activity in fish tissues. J. Aquat. Anim. Health.

[26] Ellefson WC, Richter E, Adams M, Baillies NT (1981). Evaluation of ion exchange resins and various enzymes in thiamine analysis. J. Assoc. Off. Anal. Chem..

[27] Kim CS, Bowers JA (1988). Thiamin content of chicken muscle and effect of purification variables on determined Values. Poult. Sci..

[28] Farrer K, Hollenberg W (1953). Adsorption of thiamine on glassware. Analyst.

[29] Karatapanis AE, Fiamegos YC, Stalikas CD (2010). Study of the behavior of water-soluble vitamins in HILIC on a Diol Column. Chromatographia.

[30] Brown S, Honeyfield D, Vandenbyllaardt L (1998). Thiamine analysis in fish tissues. Am. Fish. Soc. Symp..

[31] Kodamatani H, Iwaya Y, Saga M (2017). Ultra-sensitive HPLC-photochemical reaction-luminol chemiluminescence method for the measurement of secondary amines after nitrosation. Anal. Chim. Acta.

[32] Dalene M, Mathiasson L, Jönsson JÅ (1981). Trace analysis of free amines by gas–liquid chromatography. J. Chromatogr. A.

[33] Suffridge CP, Bolaños LM, Bergauer K (2020). Exploring vitamin B1 cycling and its connections to the microbial community in the North Atlantic Ocean. Front. Mar. Sci..

[34] Okbamichael M, Sañudo-Wilhelmy SA (2005). Direct determination of vitamin B1 in seawater by solid-phase extraction and high-performance liquid chromatography quantification. Limnol. Oceanogr. Methods.

[35] Suffridge C, Cutter L, Sañudo-Wilhelmy SA (2017). A new analytical method for direct measurement of particulate and dissolved B-vitamins and their congeners in seawater. Front. Mar. Sci..

[36] Seed B (2000). Silanizing glassware. Curr. Protoc. Cell Biol..

[37] Ditter D, Mahler H-C, Roehl H (2018). Characterization of surface properties of glass vials used as primary packaging material for parenterals. Eur. J. Pharm. Biopharm..

[38] Ennis RD, Pritchard R, Nakamura C (2001). Glass vials for small volume parenterals: Influence of drug and manufacturing processes on glass delamination. Pharm. Dev. Technol..

[39] Blackmer R (1990). A Study of the Chemical Resistance of Glass Ampules.

[40] How It’s Made—Laboratory Glassware. https://www.youtube.com/watch?v=QUOz4eqobVw Channel D. Accessed 17 Sept 2023.

[41] Thakker KD, Higuchi T, Sternson LA (1979). Loss of a hydrophobic amine from solution by adsorption onto container surfaces. J. Pharm. Sci..

[42] Liu X, Cheng J, Lu X, Wang R (2014). Surface acidity of quartz: Understanding the crystallographic control. Phys. Chem. Chem. Phys..

[43] Washabaugh MW, Jencks WP (1988). Thiazolium C(2)-proton exchange: Structure-reactivity correlations and the pKa of thiamin C(2)-H revisited. Biochemistry.

[44] Pérez-Caballero G, Pérez-Arévalo JF, Morales-Hipólito EA, Carbajal-Arenas ME, Rojas-Hernández A (2011). Potentiometric study of acid-base properties of thiamine hydrochloride and thiamine mononitrate in aqueous medium. J. Mex. Chem. Soc..

[45] Cain AH, Sullivan GR, Roberts JD (1977). The protonation site of vitamin B1 as determined from natural-abundance nitrogen-15 nuclear magnetic resonance spectra. JACS..

[46] Maier GD, Metzler DE (1957). Structures of thiamine in basic solution. JACS..

[47] Xu B, Liu F, Brookes PC, Xu J (2018). Microplastics play a minor role in tetracycline sorption in the presence of dissolved organic matter. Environ. Pollut..

[48] Rochman CM, Manzano C, Hentschel BT, Simonich SLM, Hoh E (2013). Polystyrene plastic: A source and sink for polycyclic aromatic hydrocarbons in the marine environment. Environ. Sci. Technol..

[49] Penner NA, Nesterenko PN, Ilyin MM, Tsyurupa MP, Davankov VA (1999). Investigation of the properties of hypercrosslinked polystyrene as a stationary phase for high-performance liquid chromatography. Chromatographia.

[50] Mayr JA, Freisinger P, Schlachter K (2011). Thiamine pyrophosphokinase deficiency in encephalopathic children with defects in the pyruvate oxidation pathway. Am. J. Hum. Genet..

[51] Pillai SA, Chobisa D, Urimi D, Ravindra N (2016). Pharmaceutical glass interactions: A review of possibilities. J. Pharm. Sci. Res..

[52] Enever RP, Po ALW, Shotton E (1977). Factors influencing decomposition rate of amitriptyline hydrochloride in aqueous solution. J. Pharm. Sci..

[53] Quarry MA, Sebastian DS, Diana F (2002). Investigation of 4,5-epoxymorphinan degradation during analysis by HPLC. J. Pharm. Biomed. Anal..

[54] Taub A, Katz IV, Katz M (1949). The stability of thiamine hydrochloride and mono-nitrate in parenteral vitamin B complex and iron solutions. J. Am. Pharm. Assoc..

[55] Huang J (2022). Effect of Metal Ions and Temperature on Stability of Thiamine Determined by HPLC.

[56] Gold K (1968). Some factors affecting the stability of thiamine. Limnol. Oceanogr..

[57] Edwards KA, Randall EA, Tu-Maung N (2019). Periplasmic binding protein-based magnetic isolation and detection of thiamine in complex biological matrices. Talanta..

[58] Sauer U, Korf U (2011). Impact of substrates for probe immobilization. Protein Microarrays: Methods and Protocols.

[59] Shah KG, Yager P (2017). Wavelengths and lifetimes of paper autofluorescence: A simple substrate screening process to enhance the sensitivity of fluorescence-based assays in paper. Anal. Chem..

[60] Porter K, Lodge JK (2021). Determination of selected water-soluble vitamins (thiamine, riboflavin, nicotinamide and pyridoxine) from a food matrix using hydrophilic interaction liquid chromatography coupled with mass spectroscopy. J. Chromatogr. B.

[61] Gill BD, Saldo SC, McGrail IJ, Wood JE, Indyk HE (2020). Rapid method for the determination of thiamine and pantothenic acid in infant formula and milk-based nutritional products by liquid chromatography-tandem mass spectrometry. J. AOAC Int..

[62] Monteverde DR, Gómez-Consarnau L, Cutter L, Chong L, Berelson W, Sañudo-Wilhelmy SA (2015). Vitamin B1 in marine sediments: Pore water concentration gradient drives benthic flux with potential biological implications. Front. Microbiol..

[63] Paerl RW, Curtis NP, Bittner MJ (2023). Use and detection of a vitamin B1 degradation product yields new views of the marine B1 cycle and plankton metabolite exchange. mBio.

[64] Gold K, Roels O, Bank H (1966). Temperature dependent destruction of thiamine in seawater. Limnol. Oceanogr..

[65] Vishniac H (1961). A biological assay for thiamine. Limnol. Oceanogr..

[66] Salgin S, Takaç S, Özdamar T (2005). A parametric study on protein-membrane-ionic environment interactions for membrane fouling. Sep. Sci. Technol..

[67] Su W, Chen C, Xu H, Yang W, Dai H (2016). Filtering whitewater with an ultrafiltration membrane: Effects of the interaction between dissolved organics and metal ions on membrane fouling. Bioresources.

[68] Schmidhalter U, Evéquoz M, Studer C, Oertli J, Kahr G (1994). Adsorption of thiamin (vitamin B1) on soils and clays. Soil Sci. Soc. Am. J..

[69] Weib, N. & Pruszkowski, S. The best material for original Eppendorf Tubes and Plates: Properties and chemical resistance of polypropylene. *Application note 056* 2013; https://online-shop.eppendorf.com/eshopdownload/downloadbykey/038853_Application-Note_186. Accessed 4 April 2017.

[70] Kilincer M, Saygin H, Ozyurek M, Baysal A (2023). Sorption of thiamin (vitamin B1) onto micro(nano)plastics: pH dependence and sorption models. J. Environ. Sci. Health A.

[71] Sannino DR, Dobson AJ, Edwards K, Angert ER, Buchon N (2018). The Drosophila melanogaster Gut microbiota provisions thiamine to its host. mBio.

[72] Sannino DR, Kraft CE, Edwards KA, Angert ER (2018). Thiaminase I provides a growth advantage by salvaging precursors from environmental thiamine and its analogs in *Burkholderia thailandensis*. Appl. Environ. Microbiol..

