# Supplementary material for: Pre-analytical challenges from adsorptive losses associated with thiamine analysis
Source: Sci Rep. 2024 May 4;14:10269. doi: 10.1038/s41598-024-60910-0 (PMC11069560; doi:10.1038/s41598-024-60910-0)
Supplement: Supplementary file 1 — Supplementary Information. [file 41598_2024_60910_MOESM1_ESM.pdf]

Pre-analytical challenges from adsorptive losses associated with thiamine analysis

Katie A. Edwards*[,1,2], Eileen A. Randall[†,3], Patricia C. Wolfe[†,1], Clifford E. Kraft[3], Esther R. Angert[2]

[1]Department of Pharmaceutical Sciences, Binghamton University, Binghamton, NY, 13902

[2]Department of Microbiology, Cornell University, Ithaca, NY, 14853

[3]Department of Natural Resources and the Environment, Cornell University, Ithaca, NY, 14853

*Corresponding author
† These authors contributed equally

Contents

Fig. S1. Concentration of thiamine recovered following container storage. 2

Fig. S2. Impact of time on losses to silanized glass vials. 3

Fig. S3. Levels of 300, 500, and 750 µL fluid in 10x75 mm and 12x75 mm borosilicate glass culture tubes 3

Table S1. Impact of Type I borosilicate glass tube dimensions on thiamine loss 4

Fig. S4. Thiamine concentration recovered from glass and plastic tubes after storage in TCA. 5

Fig. S5. Impact of pH on thiamine losses to HPLC vials 5

Fig. S6. Thiochrome recovered from plastic and glass tubes. 6

Fig. S7. Recovery of thiamine, TMP, and TDP following storage in plastic and glass containers. 7

Fig. S8. Glass and plastic filtration apparatuses 8

Fig. S9. Concentration of thiamine recovered following filtration through 47 mm membranes. 8

Filter Calculations: 9

Fig. S10. Autofluorescence and specific signals from filters 9

Table S2. Time to pass 200 mL 100 nM thiamine in deionized tap water through filters 10

Fig. S11. Effect of flow rate on thiamine concentration recovered from GF/F filters. 10

Fig. S12. Thiamine concentration (nM) recovered from polyethersulfone membrane filters. 11

Fig. S13. Concentration of thiamine recovered from river water. 12

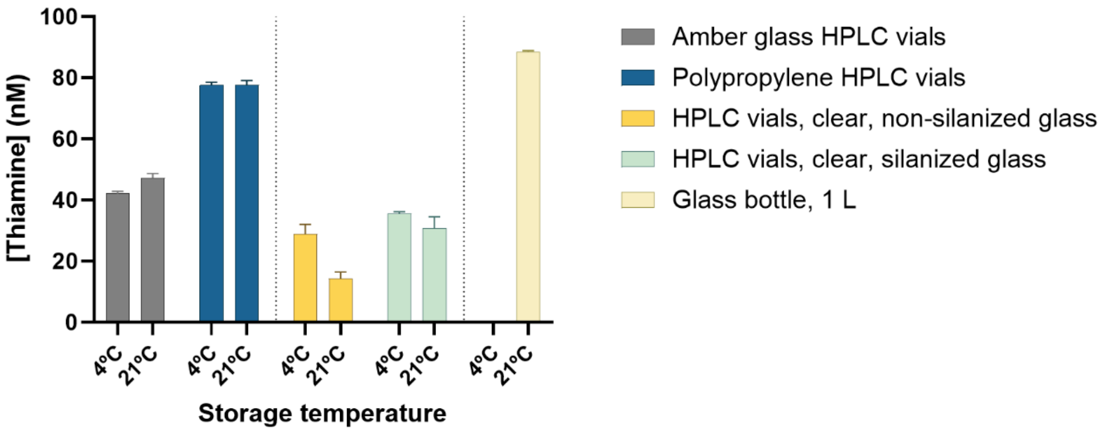

a.)

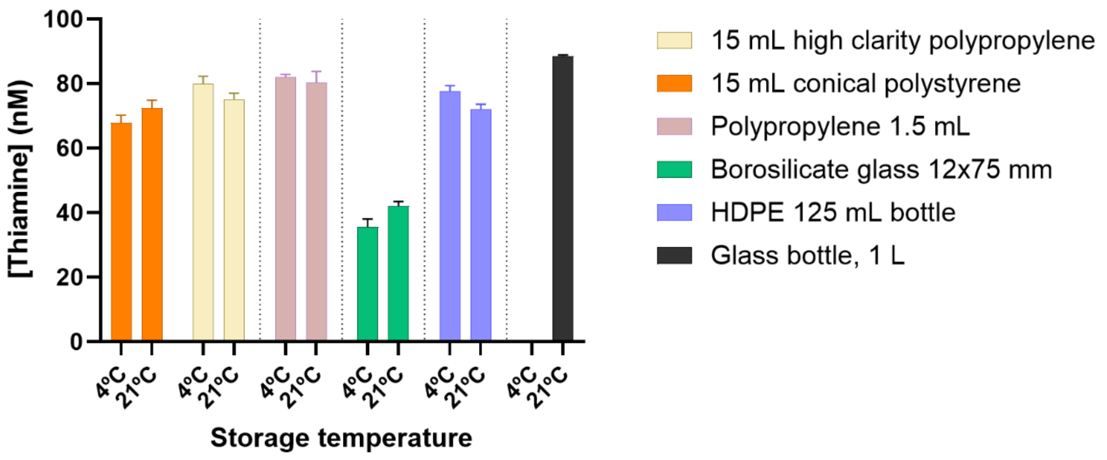

b.)

Fig. S1. Concentration of thiamine recovered following container storage. Concentration of thiamine recovered (nM) following storage of 1 mL 100 nM thiamine in deionized water for 1 hour at 4˚C or 21˚C in a.) 8x40 mm 1 mL amber glass and polypropylene autosampler vials, 1.5 mL clear non-silanized and silanized glass autosampler vials, and b.) polypropylene and polystyrene 15-mL centrifuge tubes, polypropylene 1.5 mL centrifuge tubes, glass culture tubes (10x75 mm), and high-density polyethylene 125 mL bottles as compared to a 1 L stock solution stored at 21˚C in a glass media bottle. The results are after conversion of the thiamine remaining in solution to thiochrome using alkaline ferricyanide with fluorescence detection at λ_{ex} = 360/40 nm, λ_{em} = 450/50 nm. A vertical dashed line separates materials that may be directly compared based on their surface-to-volume ratios.

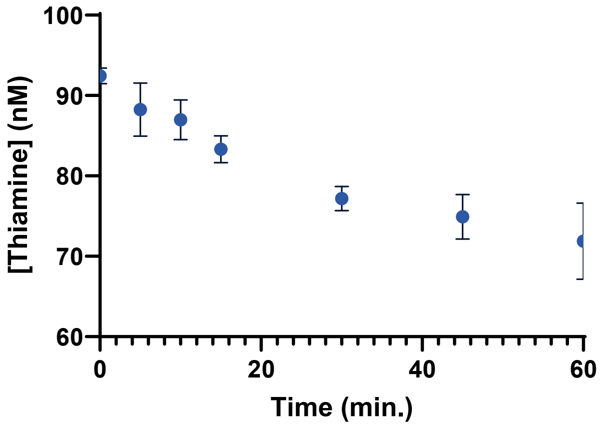

Fig. S2. Impact of time on losses to silanized glass vials. The thiamine concentration recovered following storage of 1 mL 100 nM thiamine for 0 to 60 minutes in clear 1.5 mL silanized glass HPLC vials. The results are after conversion of the thiamine remaining in solution to thiochrome using alkaline ferricyanide with fluorescence detection at λ_{ex} = 360/40 nm, λ_{em} = 450/50 nm.

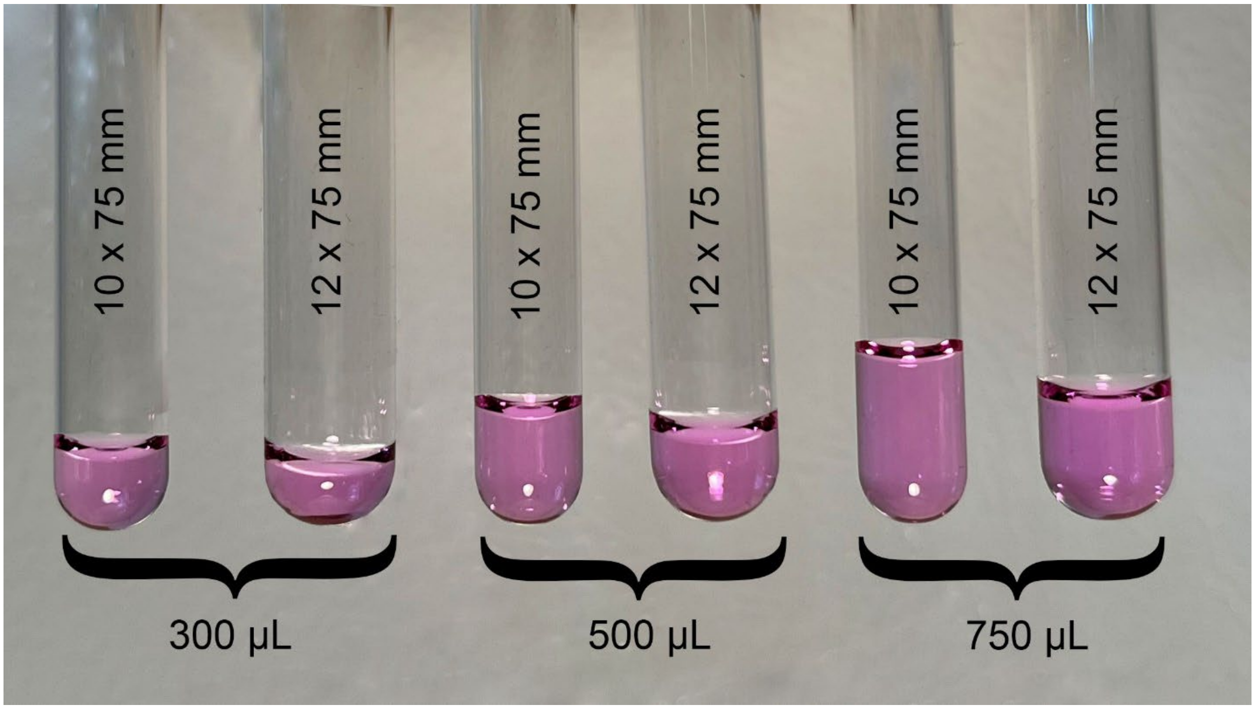

Fig. S3. Levels of 300, 500, and 750 µL fluid in 10x75 mm and 12x75 mm borosilicate glass culture tubes. For illustration purposes, a dilute sulforhodamine B solution in HPLC grade water providing a magenta color was used in this picture.

Table S1. Impact of Type I borosilicate glass tube dimensions on thiamine loss

Static					
Sample volume	Tube inner diameter	Fluid height	Inner surface area*	Surface area/ Volume	pmol thiamine lost
300 μL	8	6.5	164 mm^2	0.54 mm^2/μL	16.8
500 μL	8	10.0	252 mm^2	0.50 mm^2/μL	27.1
750 μL	8	14.0	352 mm^2	0.47 mm^2/μL	35.9
300 μL	10	4.0	157 mm^2	0.52 mm^2/μL	1.7
500 μL	10	7.0	220 mm^2	0.44 mm^2/μL	6.7
750 μL	10	11.0	345 mm^2	0.46 mm^2/μL	36.0
Vortexed					
Sample volume	Tube inner diameter	Fluid height	Inner surface area*	Surface area/ Volume	pmol thiamine lost
300 μL	8	20.0	503 mm^2	1.68 mm^2/μL	20.2
500 μL	8	25.0	629 mm^2	1.26 mm^2/μL	38.4
750 μL	8	27.0	679 mm^2	0.90 mm^2/μL	53.7
300 μL	10	20.0	628 mm^2	2.09 mm^2/μL	16.5
500 μL	10	26.0	817 mm^2	1.63 mm^2/μL	31.9
750 μL	10	32.0	1005 mm^2	1.34 mm^2/μL	58.6

*The inner surface area was calculated using the formula $2\pi rh$ for the cylinder walls in contact with the fluid, plus the formula $2\pi r^2$ that of a hemisphere for the lower rounded tube bottom. The initial thiamine concentration was 100 nM, equating to 30, 50, and 75 pmol present in the 300, 500, and 750 μL volumes, respectively.

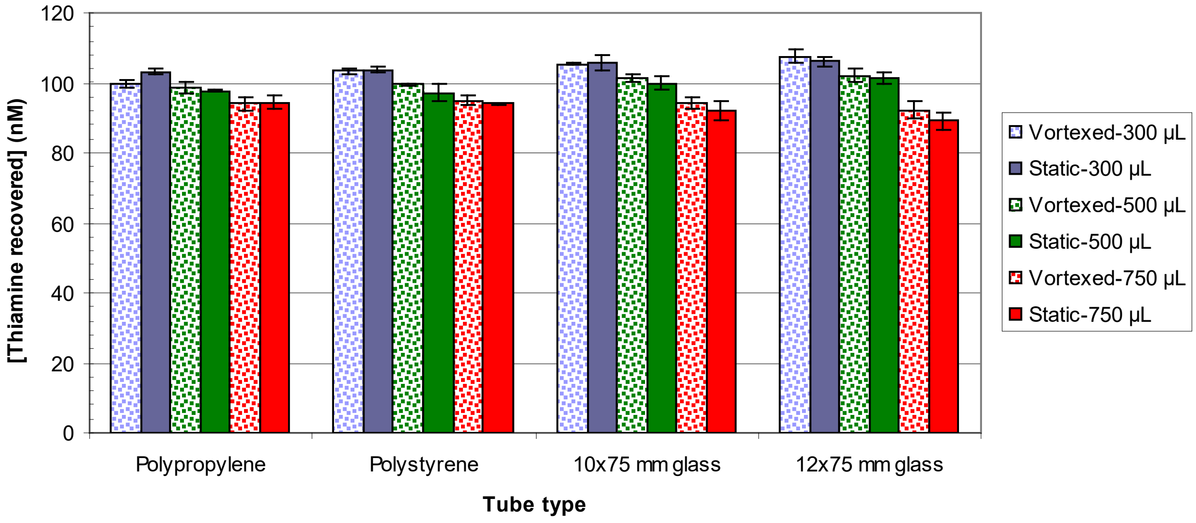

Fig. S4. Thiamine concentration recovered from glass and plastic tubes after storage in TCA. Fluorescence of 100 nM thiamine in 7.5% (w/v) TCA after storage in 10x75 mm and 12x75 mm Type I borosilicate glass tubes, 5 mL polystyrene tubes, and 1.5 mL polypropylene tubes under static or vortexed conditions for 1 hour at 21°C. The tubes were vortexed moderately every 10 minutes for 10 seconds. The results are after conversion to thiochrome using alkaline ferricyanide with detection at λ_{ex} = 360/40 nm, λ_{em} = 450/50 nm.

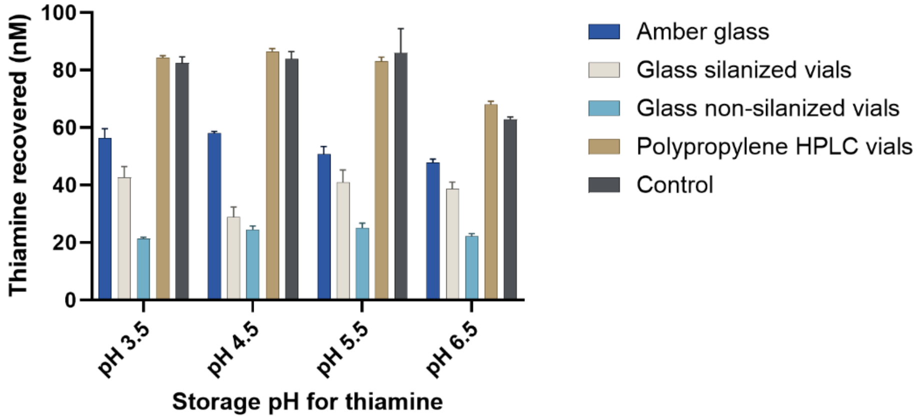

Fig. S5. Impact of pH on thiamine losses to HPLC vials. Thiamine concentration recovered following storage of 1 mL 100 nM thiamine at pH 3.5, 4.5, 5.5, and 6.5 for 1 hour at 21˚C in amber glass, clear silanized glass, clear non-silanized glass, and polypropylene 1 mL HPLC vials as compared to a 1 L stock solution stored at 21˚C in a glass media bottle (control). The results are after conversion of the thiamine remaining in solution to thiochrome using alkaline ferricyanide with fluorescence detection at λ_{ex} = 360/40 nm, λ_{em} = 450/50 nm.

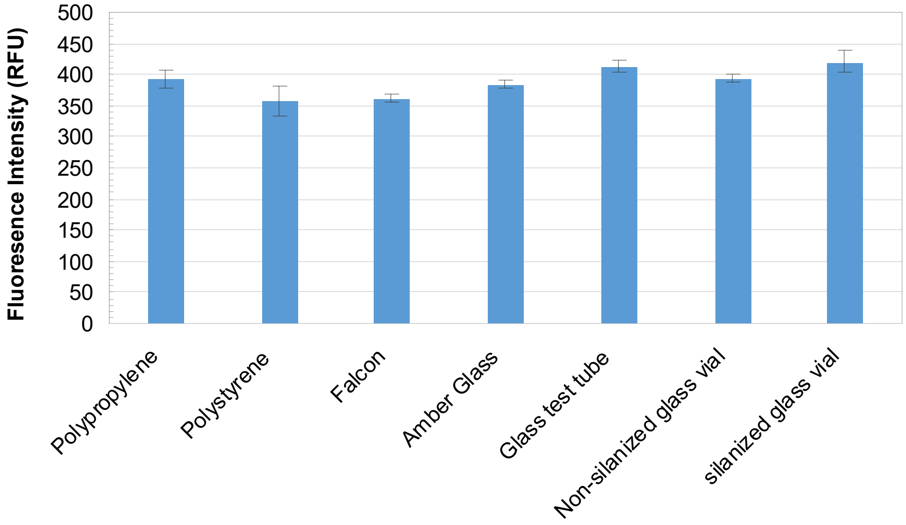

Fig. S6. Thiochrome recovered from plastic and glass tubes. Fluorescence of 100 nM thiochrome (1 mL) prepared in alkaline ferricyanide after storage in 1.5 mL microcentrifuge tubes (polypropylene), 5 mL polystyrene tubes, 15 mL polypropylene tubes, 8x40 mm amber glass, 12x75 mm Type I borosilicate glass tubes, 1.5 mL non-silanized or silanized glass autosampler vials for 1 hour at 21°C. The fluorescence of the thiochrome in the supernatant was read directly with detection at λ_{ex} = 360/40 nm, λ_{em} = 450/50 nm.

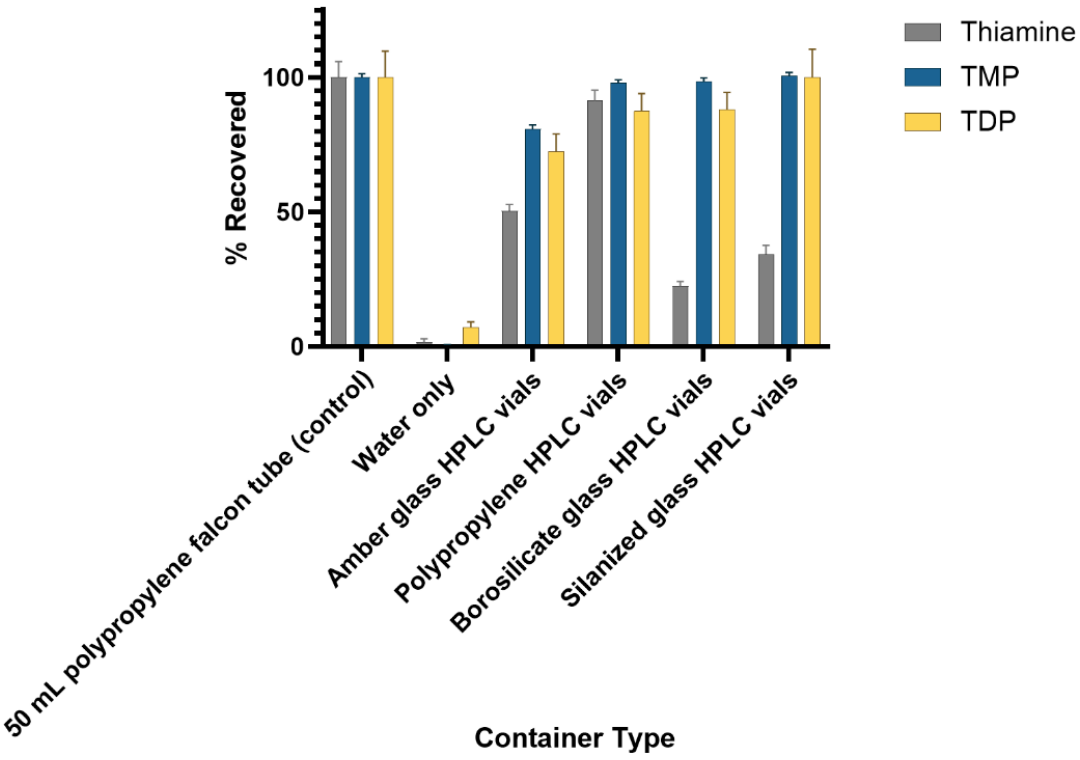

a.)

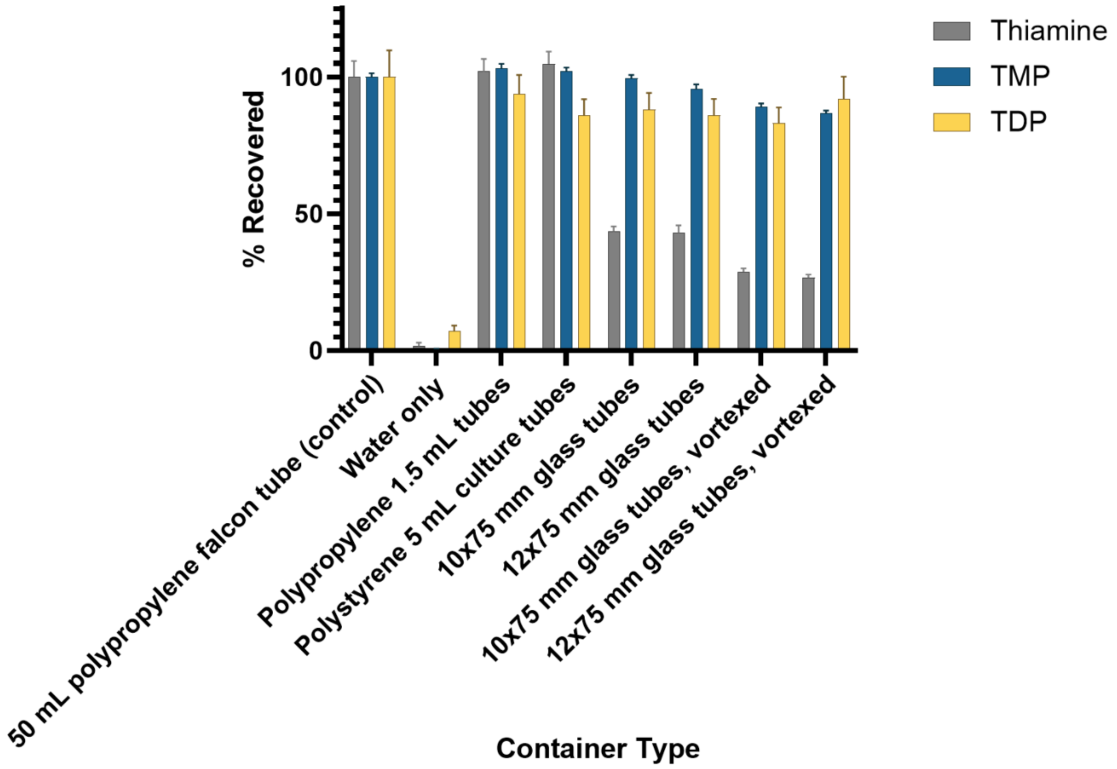

b.)

Fig. S7. Recovery of thiamine, TMP, and TDP following storage in plastic and glass containers. Recovery of thiamine in the solution following storage of 1 mL 100 nM thiamine, TMP, or TDP for 3 hours at 21˚C in HPLC grade water. A.) Amber glass, clear non-silanized, clear silanized glass, and polypropylene HPLC vials b.) polypropylene and polystyrene 15-mL centrifuge tubes, polypropylene 1.5 mL centrifuge tubes, glass culture tubes (10x75 mm), and high-density polyethylene 125 mL bottles were compared to the stock solution stored at 21˚C in a 50 mL polypropylene tube. The results are taken from the solutions after conversion to thiochrome using alkaline ferricyanide with detection in a microplate reader at λ_{ex} = 360/40 nm, λ_{em} = 450/50 nm.

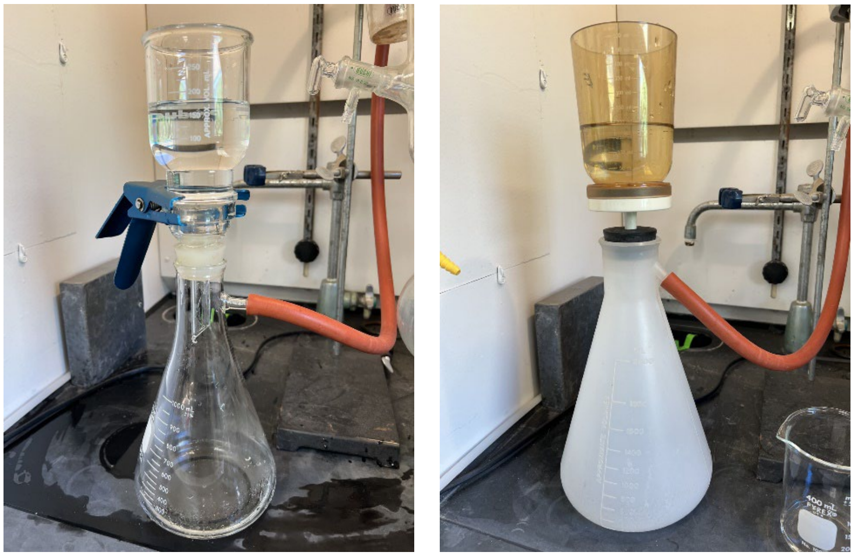

Fig. S8. Glass and plastic filtration apparatuses

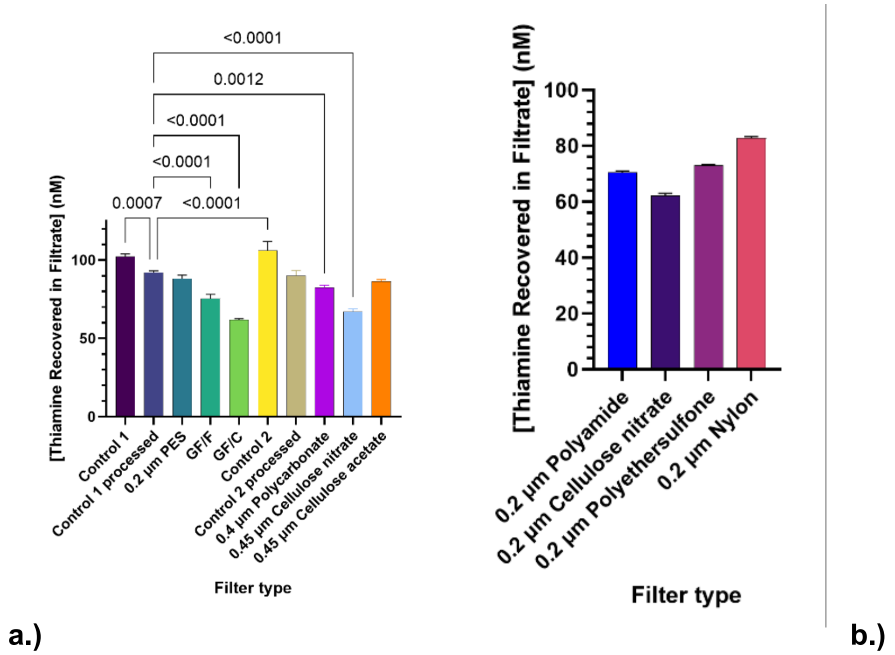

Fig. S9. Concentration of thiamine recovered following filtration through 47 mm membranes. The concentration of thiamine (nM) recovered in the filtrate from 200 mL 100 nM thiamine in deionized water passed through a.) 0.2 µm 47 mm polyethersulfone (PES), glass fiber (GF/F and GF/C), 0.4 µm polycarbonate, 0.45 µm cellulose nitrate, and 0.45 µm cellulose acetate membranes and b.) 0.2 µm 47 mm polyamide, cellulose nitrate, polyethersulfone, and nylon membranes. The control was the 1 L solution stored in a.) a polypropylene graduated cylinder and b.) a glass Pyrex bottle. The processed control was the glass filter housing and collection flask only, without a filter. The results are after conversion of the thiamine remaining in solution to thiochrome using alkaline ferricyanide with fluorescence detection at λ_{ex} = 360/40 nm, λ_{em} = 450/50 nm. Error bars represent one standard deviation of triplicate thiochrome determinations

of the filtrate. A one-way ANOVA was used to compare the results for filter recovery to the processed control, with the p values listed in the figure.

Filter Calculations:

The filter calculations were done as follows: Thiamine standards were prepared (0-1000 nM) and a 50 µL volume was added to microwell plates, then converted to thiochrome using 100 µL alkaline ferricyanide for a total volume of 150 µL. The filters were pulled dry under vacuum and homogenized in 2 mL alkaline ferricyanide and 100 µL added to the wells, along with 50 µL water for a total volume of 150 µL. The volume and volume ratio of alkaline ferricyanide to aqueous solution was the same between samples and standards. The calibration curves and sample data were processed on the basis of pmol thiamine to account for the volume differences of sample to standard for the filter samples, then converted to concentrations.

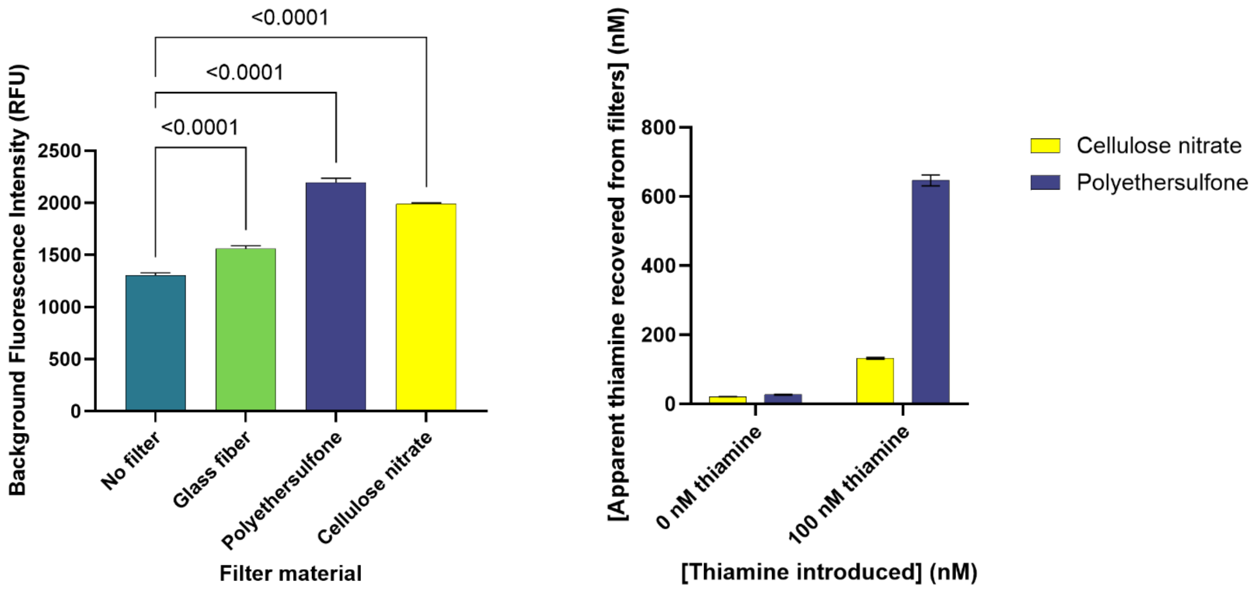

a.) b.)

Fig. S10. Autofluorescence and specific signals from filters A.) raw fluorescence intensities of 47 mm GF/F glass fiber, polyethersulfone (PES), and cellulose nitrate filters homogenized in 2 mL alkaline ferricyanide following passage of water in the absence of thiamine. Water only treated with alkaline ferricyanide is included as a no-filter control. A one-way ANOVA was used to compare the results for filter recovery to the no-filter control, with the p values listed in the figure. B.) Blank (0 nM) versus 100 nM thiamine through cellulose nitrate and PES filters. Apparent recovery of thiamine from the filtrate of 200 mL deionized water containing 0 or 100 nM added thiamine following passage through 0.2 µm 47 mm PES or cellulose nitrate membranes. The results are after conversion of the thiamine remaining in solution to thiochrome using alkaline ferricyanide with fluorescence detection at λ_{ex} = 360/40 nm, λ_{em} = 450/50 nm. Error bars represent one standard deviation of triplicate thiochrome determinations of the filtrate.

Table S2. Time to pass 200 mL 100 nM thiamine in deionized tap water through filters when vacuum was set at 600 mm Hg

**Membrane**	**Pore size**	**Time**
Whatman GF/F	0.7 μm	50 sec.
Whatman GF/C	1.2 μm	30 sec.
Millipore PES	0.22 μm	2 min. 52 sec.
Millipore Isopore HTTP	0.4 μm	2 min. 50 sec.
Whatman cellulose acetate	0.45 μm	2 min. 18 sec.
Whatman cellulose nitrate	0.45 μm	2 min. 50 sec.
Whatman polyamide	0.2 μm	4 min. 25 sec.
Whatman cellulose nitrate	0.2 μm	4 min. 11 sec.
Whatman nylon	0.2 μm	5 min. 42 sec.
Millipore PES	0.22 μm	2 min. 43 sec.
No membrane		20 sec.

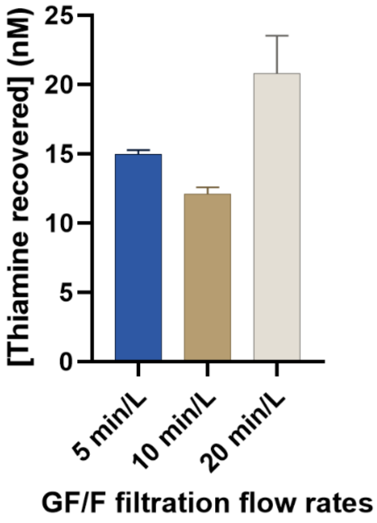

Fig. S11. Effect of flow rate on thiamine concentration recovered from GF/F filters. A 1 L solution of regular tap water containing 100 pM thiamine was filtered at 5 to 20 min./L through GF/F filters. The results are after conversion of the thiamine remaining in solution to thiochrome using alkaline ferricyanide with fluorescence detection at λ_{ex} = 360/9 nm, λ_{em} = 450/15 nm.

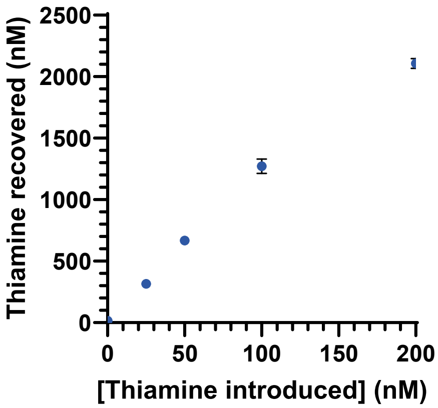

Fig. S12. Thiamine concentration (nM) recovered from polyethersulfone membrane filters. Recovery of thiamine following filtration of 125 mL 0-200 nM thiamine through 0.2 µm PES membranes. The results are after conversion of the thiamine captured on the filters to thiochrome using alkaline ferricyanide with fluorescence detection at λ_{ex} = 360/9 nm, λ_{em} = 450/15 nm.

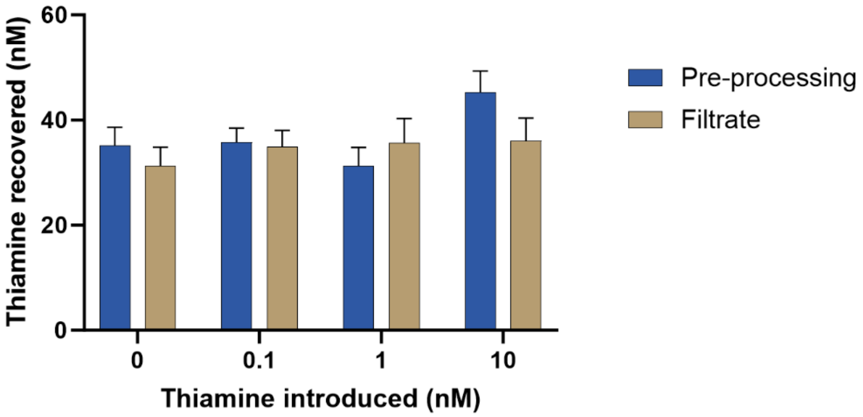

a.)

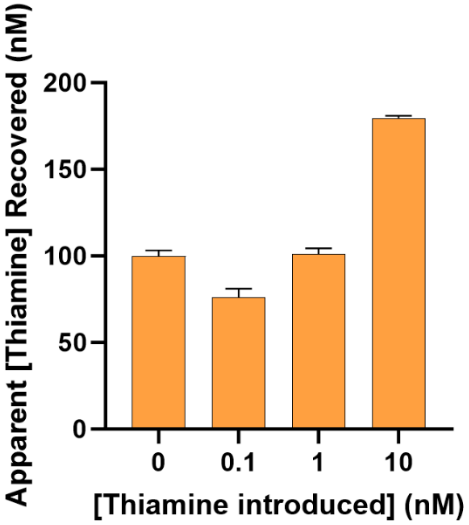

b.)

Fig. S13. Concentration of thiamine recovered from river water. Recovery of thiamine spiked at 0-10 nM in water collected from the Susquehanna River, Binghamton, NY following filtration of 250 mL through 0.7 μm GF/F filters. a.) water before and after filtration b.) apparent thiamine recovered from the GF/F filters. The results are after conversion of the thiamine remaining in solution to thiochrome using alkaline ferricyanide with fluorescence detection at λ_{ex} = 360/9 nm, λ_{em} = 450/15 nm.
